# Pan-American Guidelines for the treatment of SARS-CoV-2/COVID-19: a joint evidence-based guideline of the Brazilian Society of Infectious Diseases (SBI) and the Pan-American Association of Infectious Diseases (API)

**DOI:** 10.1186/s12941-023-00623-w

**Published:** 2023-08-07

**Authors:** Alexandre Naime Barbosa, Alberto Chebabo, Carlos Starling, Clevy Pérez, Clóvis Arns Cunha, David de Luna, Estevão Portela Nunes, Gabriela Zambrano, Juliana Carvalho Ferreira, Julio Croda, Maicon Falavigna, Monica Maria Gomes-da-Silva, Monica Thormann, Sergio Cimerman, Suena Medeiros Parahiba, Suzana Tanni, Wanderley Marques Bernardo, Alfonso J. Rodriguez-Morales

**Affiliations:** 1 Infectious Diseases Department - Botucatu School of Medicine - UNESP, Av. Prof. Mário R. G. Montenegro, s/n, Botucatu, SP CEP 18.618-687 Brazil; 2 Universidade Federal do Rio de Janeiro, Avenida Professor Rodolpho Paulo Rocco, 255, 50. Andar, Rio de Janeiro, RJ CEP 21941-913 Brazil; 3 Brazilian Society for Infectious Diseases, Rua Teixeira da Silva, 660, São Paulo, SP CEP 04002-033 Brazil; 4 Sociedade Mineira de Infectologia - SMI, Avenida João Pinheiro, 161, Belo Horizonte, MG CEP 30130-180 Brazil; 5 Universidad Autónoma de Santo Domingo (UASD), Avenida Simón Bolívar, 902, Santo Domingo, 10108 República Dominicana; 6 Universidade Federal do Paraná, Rua XV de Novembro, 1299, Curitiba, PR CEP 80060-000 Brazil; 7 Comisión Nacional de Arbitraje Médico, C Mitla, 250, Ciudad de México, 03020 México; 8 Instituto Nacional de Infectologia (INI), Fiocruz, Avenida Brasil, 4365, Rio de Janeiro, RJ CEP 21040-360 Brazil; 9 Faculty of Medicine, Department of Infectious Diseases, Universidad Central del Ecuador, Quito, Ecuador; 10 Divisão de Pneumologia, Instituto do Coração, Hospital das Clínicas da Faculdade de Medicina da Universidade de São Paulo, Universidade de São Paulo, Avenida Dr. Enéas Carvalho de Aguiar, 44, São Paulo, SP CEP 05403-900 Brazil; 11 Intensive Care Unit, AC Camargo Cancer Center, Rua Prof. Antônio Prudente, 211, São Paulo, SP CEP 01509-001 Brazil; 12 Oswaldo Cruz Foundation, Avenida Costa e Silva, s/n, Cidade Universitária, Campo Grande, MS CEP 79070-900 Brazil; 13 HTAnalyze Consulting and Training, Rua João Abbott, 109, Porto Alegre, RS CEP 90460-150 Brazil; 14 Infectious Disease Control Service, Clinical Hospital, Universidade Federal Do Paraná, Rua General Carneiro, 181, Curitiba, PR CEP 80060-900 Brazil; 15 Hospital Salvador Bienvenido Gautier, Calle Alexander Fleming, 177, Santo Domingo, 10514 Dominican Republic; 16 Institute of Infectious Diseases Emilio Ribas, Avenida Dr. Arnaldo, 165, São Paulo, SP CEP 05402-000 Brazil; 17 Universidade Estadual Paulista, Julio de Mesquita Filho, Distrito de Rubiao Jr, s/n, Botucatu, SP CEP 18618-970 Brazil; 18 Medical Education Development Center (CEDEM) of Medical Faculty of São Paulo University (FMUSP), São Paulo, SP Brazil; 19 Grupo de Investigación Biomedicina, Faculty of Medicine, Fundación Universitaria Autónoma de Las Américas-Institución Universitaria Visión de Las Américas, 660003 Pereira, Risaralda Colombia; 20 Clinical Epidemiology and Biostatistics, Faculty of Health Sciences, Universidad Científica del Sur, Lima, 4861 Peru; 21 Gilbert and Rose-Marie Chagoury School of Medicine, Lebanese American University, P.O. Box 36, Beirut, Lebanon; 22 Latin American Network of Coronavirus Disease 2019 – COVID-19 Research (LANCOVID-19), Pereira, Risaralda Colombia; 23 Pontificia Universidad Católica del Ecuador, Facultad de Medicina, Posgrado de Medicina Interna, Quito, Ecuador

**Keywords:** COVID-19, SARS-CoV-2, Therapy, Guidelines, Treatment

## Abstract

**Background:**

Since the beginning of the COVID-19 pandemic, therapeutic options for treating COVID-19 have been investigated at different stages of clinical manifestations. Considering the particular impact of COVID-19 in the Americas, this document aims to present recommendations for the pharmacological treatment of COVID-19 specific to this population.

**Methods:**

Fifteen experts, members of the Brazilian Society of Infectious Diseases (SBI) and the Pan-American Association of Infectious Diseases (API) make up the panel responsible for developing this guideline. Questions were formulated regarding prophylaxis and treatment of COVID-19 in outpatient and inpatient settings. The outcomes considered in decision-making were mortality, hospitalisation, need for mechanical ventilation, symptomatic COVID-19 episodes, and adverse events. In addition, a systematic review of randomised controlled trials was conducted. The quality of evidence assessment and guideline development process followed the GRADE system.

**Results:**

Nine technologies were evaluated, and ten recommendations were made, including the use of tixagevimab + cilgavimab in the prophylaxis of COVID-19, tixagevimab + cilgavimab, molnupiravir, nirmatrelvir + ritonavir, and remdesivir in the treatment of outpatients, and remdesivir, baricitinib, and tocilizumab in the treatment of hospitalised patients with severe COVID-19. The use of hydroxychloroquine or chloroquine and ivermectin was discouraged.

**Conclusion:**

This guideline provides recommendations for treating patients in the Americas following the principles of evidence-based medicine. The recommendations present a set of drugs that have proven effective in the prophylaxis and treatment of COVID-19, emphasising the strong recommendation for the use of nirmatrelvir/ritonavir in outpatients as the lack of benefit from the use of hydroxychloroquine and ivermectin.

**Supplementary Information:**

The online version contains supplementary material available at 10.1186/s12941-023-00623-w.

## Background

The increased number of severe cases of viral pneumonia caused by the Severe Acute Respiratory Syndrome Coronavirus 2 (SARS-CoV-2) in China in 2019 and its worldwide spread led the World Health Organization (WHO) to declare the Coronavirus Disease 2019 (COVID-19) a pandemic on March 11, 2020, persisting as public health emergency of international concern up to May 5, 2023 [1, 2]. As of August 2023, more than 768.98 million confirmed cases and more than 6.95 million deaths from COVID-19 have been reported worldwide [3]. According to the WHO, more than 193.21 million cases have been recorded in the Americas, and the continent has the highest COVID-19 death numbers in the world by region, with 2,958,858 cases with fatal outcome [4]. These figures are due to the high incidence of cases and deaths in the largest countries in the Americas. The United States of America (USA) has recorded more than 103.44 million cases and 1.13 million deaths, followed by Brazil with more than 37.7 million cases and 704,659 deaths, which is then followed by Argentina with more than 10.04 million cases and 130,472 deaths, and Mexico with more than 7.63 million cases and 334,336 deaths, among others [3]. These rates have made COVID-19 a severe public health threat worldwide and in Latin America [5].

Since the beginning of the COVID-19 pandemic, the global scale of SARS-CoV-2 infection has risen considerably over time and with regional variation [6]. Numerous drugs related to the pathogenesis of SARS-CoV-2, such as those with antiviral and immunomodulatory effects and inhibitors of the inflammatory cascade, have been proposed to minimise damage in patients with suspected or some degree of infection, with promising results, particularly in high-risk populations. This group includes individuals older than 65, individuals with obesity, cardiovascular or metabolic disease, or immunocompromising conditions, and individuals who are unvaccinated or under-vaccinated [7]. In addition, the overall increase in vaccination coverage has led to a substantial drop in the risk of hospitalisation and death [7]. However, increased transmissibility of new variants of concern would still result in a rise in cases leading to excessive hospitalisations associated with COVID-19 and its complications [8].

In light of new evidence, changes in the pandemic scenario and heterogeneity in clinical practice, it is necessary to evaluate the existing evidence and formulate recommendations so that health professionals can provide adequate treatment.

## Methods

The guideline development group consisted of a group of coordinators, including one specialist in the proposed topic (ANB) and two methodologists (JCF, ST), and an expert committee (panel members), including experts from institutions of Brazil, Colombia, Ecuador, Peru, and the Dominican Republic who represent the Brazilian Society of Infectious Diseases (*Sociedade Brasileira de Infectologia*, SBI) and the Pan-American Association of Infectious Diseases (*Asociación Panamericana de Infectología*, API). Videoconferencing and face-to-face recommendation meetings, including asynchronous written communication (i.e. e-mail), were held from May 27, 2022, to July 6, 2022. A final meeting, on-site and virtual, was held from Sao Paulo, Brazil, on February 3 and 4, 2023 to conclude the basis of the current document. The guideline development process followed the Grading of Recommendations Assessment, Development, and Evaluation (GRADE) system for assessing evidence and developing recommendations [9, 10].

The expert committee formulated ten questions related to the pharmacological treatment of COVID-19 according to the PICO framework (patients, intervention, comparator, and outcome). The outcomes of interest were defined a priori and classified as critical, important, or unimportant. Only critical and important outcomes were used for making the recommendations (Table 1).

**Table 1:** Guideline questions and outcomes of importance

Question	Critical outcomes	Important outcomes
1. Should tixagevimab + cilgavimab be recommended for pre-exposure prophylaxis in people at high risk of developing severe COVID-19?	Symptomatic COVID-19 Adverse event with death	Serious adverse event
2. Should certain (see bottom of table) monoclonal antibodies be recommended for outpatients with mild COVID-19?^a^	Hospitalisation Death	Serious adverse event
3. Should molnupiravir be recommended for outpatients with mild COVID-19?	Hospitalisation Death	Serious adverse event
4. Should nirmatrelvir/ritonavir be recommended for outpatients with mild COVID-19?	Hospitalisation Death	Serious adverse event
5. Should remdesivir be recommended for outpatients with mild COVID-19?	Hospitalisation Death	Serious adverse event
6. Should hydroxychloroquine or chloroquine be recommended for outpatients with mild COVID-19?	Hospitalisation Death	Serious adverse event
7. Should ivermectin be recommended for outpatients with mild COVID-19?	Hospitalisation Death	Serious adverse event
8. Should remdesivir be recommended for hospitalised patients with severe COVID-19?	Mechanical ventilation Death	Serious adverse event
9. Should baricitinib be recommended for hospitalised patients with severe COVID-19?	Mechanical ventilation Death	Serious adverse event
10. Should tocilizumab be recommended for hospitalised patients with severe COVID-19?	Mechanical ventilation Death	Serious adverse event

^a^In this question, the following monoclonal antibodies were considered: bamlanivimab + etesevimab, casirivimab + imdevimab, sotrovimab, bebtelovimab, and tixagevimab + cilgavimab. During the panel, members decided not to make recommendations for bamlanivimab, casirivimab, etesevimab, imdevimab, regdanvimab, and sotrovimab due to a lack of evidence of effectiveness in the scenario of omicron variant circulation and for bebtelovimab due to lack of evidence of effectiveness

### Evidence search and synthesis

A team of experienced methodologists searched and synthesised evidence independent of the expert committee.

Searches were performed on MEDLINE, Embase, ClinicalTrials.gov and Google Scholar databases. The search strategy was restricted to phase III randomised controlled trials (RCTs), with keywords pre-established by the specialist coordinators, without limitations on language or publication date (Additional file 1: Table S1).

Two researchers independently screened titles and abstracts. If an abstract was considered relevant, the paper was included for full-text review to confirm eligibility. The reasons for inclusion or exclusion were recorded and presented according to the recommendations of the Preferred Reporting Items for Systematic Reviews and Meta-Analyses (PRISMA) (Additional file 1: Figs S1–S10). Then, two reviewers independently abstracted the data from selected studies and performed meta-analyses whenever possible. The risk of bias was assessed using an adapted version of the Cochrane Risk of Bias Tool 2.0. Finally, the quality of evidence was assessed using GRADE (Table 2).

**Table 2:** Levels of evidence according to the Grading of Recommendations Assessment, Development, and Evaluation (GRADE)

Level	Definition	Implications
High (⨁⨁⨁⨁)	We are very confident that the true effect lies close to that of the estimate of the effect	Future research is unlikely to change confidence in the estimated effect
Moderate (⨁⨁⨁O)	We are moderately confident in the effect estimate: the true effect is likely to be close to the estimate of the effect, but there is a possibility that it is substantially different	Future research will likely have a major impact on confidence in the estimated effect and may change this estimate
Low (⨁⨁OO)	Our confidence in the effect estimate is limited: the true effect may be substantially different from the estimate of the effect	Future research will likely have a major impact on confidence in the estimated effect and will likely change this estimate
Very low (⨁OOO)	We have very little confidence in the effect estimate: the true effect is likely to be substantially different from the estimate of the effect	Any estimate of an effect is very uncertain

Adapted from: Grading of Recommendations Assessment, Development, and Evaluation (GRADE) Working Group. Handbook for grading the quality of evidence and the strength of recommendations using the GRADE approach. Updated October 2013. Available from: https://gdt.gradepro.org/app/handbook/handbook.html [11]

### Development of recommendations

On May 27, 2022, a recommendation meeting was held in São Paulo, Brazil, in a hybrid format (in person and remote). In the meeting, each question with the underlying evidence was presented to the panel of experts to develop recommendations. Before starting the meeting, all experts and methodologists declared and signed their relevant conflicts of interest pertinent to each of the 10 guideline questions. A second virtual meeting was required to finalise the process, held on July 6, 2022.

The GRADE Evidence to Decision (EtD) framework was used to evaluate the priority of the problem, the magnitude of undesirable effects, evidence of benefits and risks, quality of evidence, costs and use of resources, feasibility, and aspects related to equity, patient values and preferences, and acceptability. Finally, the panel made a recommendation, where the direction of the course of action was discussed (whether to recommend or not to recommend the use of the intervention), and the strength of recommendation was defined as strong or conditional according to the GRADE system (Table 3). The terminology "we recommend" and "we suggest" denote different degrees of emphasis on the strength of recommendation, as follows: "We recommend" represents a strong recommendation, which should be incorporated as a routine practice, either for or against the use of a given intervention; "We suggest" represents a conditional recommendation, which applies to most situations, but due either to the lack of robust evidence or to the expected variation in treatment effectiveness, other approaches may be justifiable.

**Table 3:** Implications of the strength of recommendation for clinicians, patients, and policymakers

Target audience	Strong	Conditional
Policymakers	The recommendation should be adopted as a health care policy in most situations	Substantial debate is required, with the involvement of stakeholders
Clinicians	Most patients should receive the recommended intervention	The health professional should acknowledge that different choices may be appropriate for individual patients and should help them make decisions consistent with their values and preferences
Patients	Most individuals would want the intervention to be recommended, and only a small number would not accept this recommendation	Most individuals would want the intervention to be recommended, although a considerable number would not accept this recommendation

Source: Adapted from Grading of Recommendations Assessment, Development, and Evaluation (GRADE) Working Group. Handbook for grading the quality of evidence and the strength of recommendations using the GRADE approach. Updated October 2013. Available from: [11]

Members with a direct financial conflict of interest related to a given intervention did not vote for the related questions. The list of participants, their role in the guideline, and statement of conflicts of interest are provided in additional material (Additional file 1: Table S2).

## Results

**Fig. 1:**
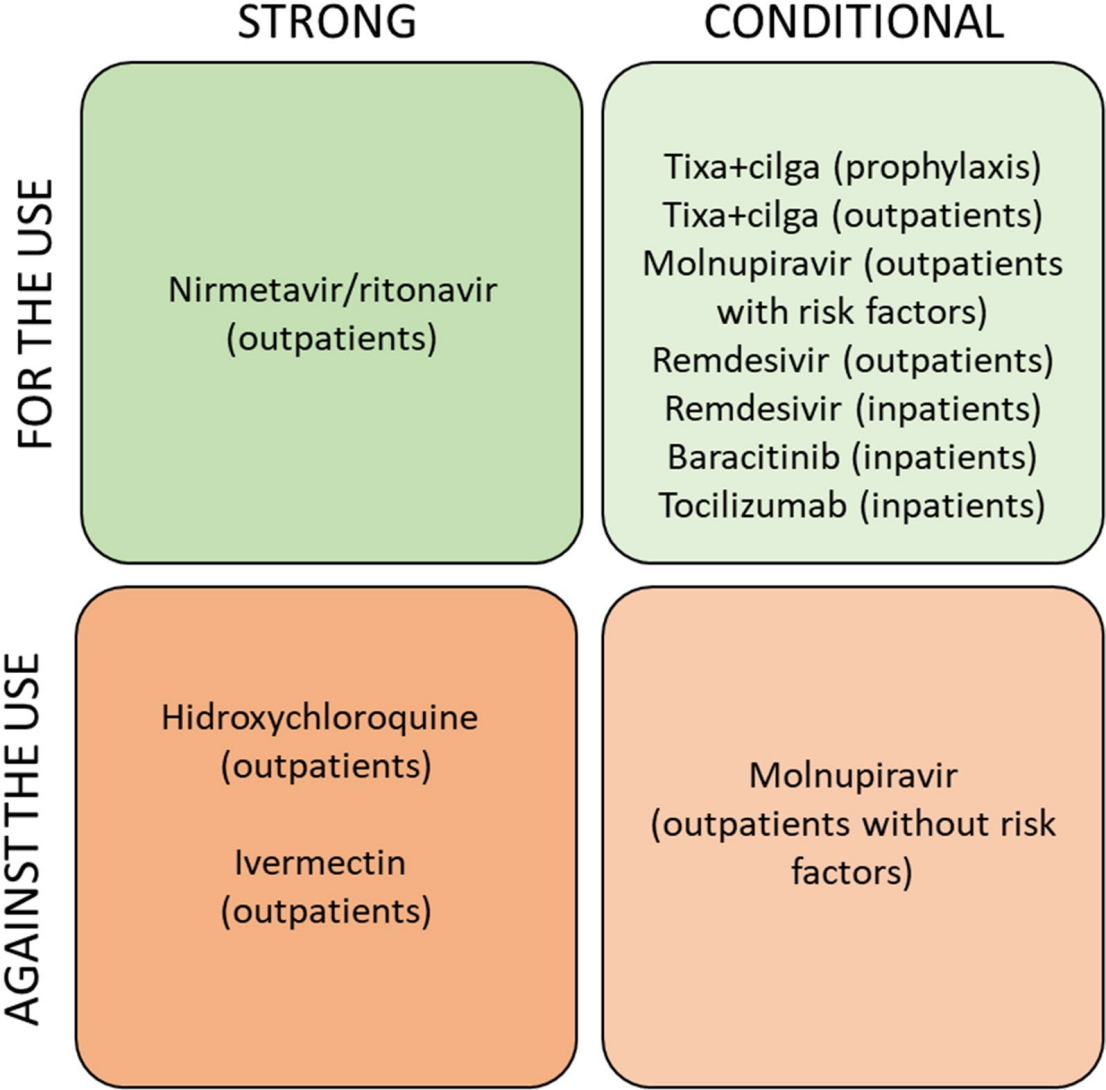
Summary of recommendations for the pharmacological treatment of COVID-19. Tixa + cilga stands for tixagevimab + cilgavimab. Source: manuscript’ authors

Ten recommendations were made. The guideline panel recommendations are summarised in Table 4 and Fig. 1. Each recommendation with a summary of the underlying evidence is presented below. In addition, detailed information regarding the evidence supporting each recommendation is shown in Additional file 1.

**Table 4:** Summary of recommendations

Recommendation 1:	We suggest using tixagevimab + cilgavimab for prophylaxis in people at high risk of developing severe COVID-19 (conditional recommendation, very low certainty in evidence)
Recommendation 2:	We suggest using tixagevimab + cilgavimab in outpatients with mild COVID-19 (conditional recommendation, moderate certainty in evidence)
Recommendation 3.1:	We suggest against using molnupiravir in outpatients with mild COVID-19 and no risk factors for severe disease (conditional recommendation, very low certainty in evidence)
Recommendation 3.2:	We suggest using molnupiravir in outpatients with mild COVID-19 and risk factors for severe disease (conditional recommendation, very low certainty in evidence)
Recommendation 4:	We recommend using nirmatrelvir/ritonavir in outpatients with mild COVID-19 (strong recommendation, moderate certainty in evidence)
Recommendation 5:	We suggest using remdesivir in outpatients with mild COVID-19 (conditional recommendation, low certainty in evidence)
Recommendation 6:	We recommend against using hydroxychloroquine or chloroquine in outpatients with mild COVID-19 (strong recommendation, moderate certainty in evidence)
Recommendation 7:	We recommend against using ivermectin in outpatients with mild COVID-19 (strong recommendation, moderate certainty in evidence)
Recommendation 8:	We suggest using remdesivir in hospitalised patients with severe COVID-19 (conditional recommendation, low certainty in evidence)
Recommendation 9:	We suggest using baricitinib in hospitalised patients with severe COVID-19 (conditional recommendation, moderate certainty in evidence)
Recommendation 10:	We suggest using tocilizumab in hospitalised patients with severe COVID-19 (conditional recommendation, moderate certainty in evidence)

### COVID-19 prophylaxis

*Recommendation 1*: we suggest using tixagevimab + cilgavimab for prophylaxis in people at high risk of developing severe COVID-19 (conditional recommendation, very low certainty in evidence).

Summary of evidence: the review identified 13 references, and one RCT (Levin et al.) evaluating the effectiveness of tixagevimab + cilgavimab in the population of interest was included [12]. The trial tested a monoclonal-antibody combination of tixagevimab and cilgavimab (AZD7442). A single 300 mg dose of AZD7442 (two consecutive 1.5 mL intramuscular injections, one containing tixagevimab and the other containing cilgavimab) was administered on day 1. Compared with placebo, tixagevimab + cilgavimab reduced the occurrence of symptomatic COVID-19 by 2% (one RCT, n = 5197, absolute risk difference of 2.0%; 95% CI − 2.7% to − 1.1%; very low certainty in evidence). No significant difference was observed for adverse events.

### Treatment of outpatients with COVID-19

*Recommendation 2*: we suggest using tixagevimab + cilgavimab in outpatients with mild COVID-19 (conditional recommendation, moderate certainty in evidence).

Summary of evidence: the review identified 53 references, and one RCT (Montgomery et al.) evaluating the effectiveness of tixagevimab + cilgavimab in the population of interest was included [13]. The trial tested the intramuscular administration of a single tixagevimab-cilgavimab 600 mg dose (two consecutive 3 mL intramuscular injections, one containing tixagevimab and the other containing cilgavimab) on day 1. Compared with placebo, tixagevimab + cilgavimab reduced hospitalisation by 5.1% (one RCT, n = 903, absolute risk difference of − 5.1%; 95% CI − 8.2% to − 1.9%; moderate certainty in evidence). No significant difference was observed for mortality or adverse events.

*Recommendation 3.1*: we suggest against using molnupiravir in outpatients with mild COVID-19 and no risk factors for severe disease (conditional recommendation, very low certainty in evidence).

*Recommendation 3.2.* We suggest using molnupiravir in outpatients with mild COVID-19 and risk factors for severe disease (conditional recommendation, very low certainty in evidence).

Summary of evidence: the review identified 26 references and one RCT (MOVe-OUT study) evaluating the effectiveness of molnupiravir in outpatients with mild COVID-19 and no risk factors for severe disease and one RCT (Tippabhotla et al.) assessing the effectiveness of molnupiravir in the population of interest were included [14, 15]. Both trials tested the oral administration of 800 mg of molnupiravir twice daily for five days in addition to standard-of-care treatment. In patients without risk factors for severe disease, no significant difference was observed for molnupiravir as compared with placebo in hospitalisation (one RCT, n = 1220, absolute risk difference of − 1.0%; 95% CI − 2.0% to 0.0%; moderate certainty in evidence), mortality (absolute risk difference of 0.0%; 95% CI, −0.0% to 0.0%; very moderate certainty in evidence), or serious adverse events (absolute risk difference of − 0.0%; 95% CI − 4.0% to 3.0%; moderate certainty in evidence) [14]. In patients with risk factors for severe disease, molnupiravir, as compared with placebo, reduced mortality (one RCT, n = 1433, absolute risk difference of − 1.0%; 95% CI − 2.0% to − 0.0%; high certainty in evidence) but did not reach statistical significance for hospitalisation (one RCT, n = 1433, absolute risk difference of − 2.0%; 95% CI − 4.0% to 1.0%; high certainty in evidence). Molnupiravir did not increase serious adverse events (one RCT, n = 1433, absolute risk difference of − 3.0%; 95% CI − 5.0% to 0.0%; high certainty in evidence) [15].

*Recommendation 4*: we recommend using nirmatrelvir/ritonavir in outpatients with mild COVID-19 (strong recommendation, moderate certainty in evidence).

Summary of evidence: the review identified 19 references, and one RCT (EPIC-HR study) evaluating the effectiveness of nirmatrelvir/ritonavir in the population of interest was included [16]. The trial assessed the administration of nirmatrelvir (300 mg) plus ritonavir (100 mg) twice daily for five days. As compared with placebo, nirmatrelvir/ritonavir reduced mortality (one RCT, n = 2246, absolute risk difference of − 1.0%; 95% CI − 1.6% to − 0.4%; moderate certainty in evidence) and hospitalisation (one RCT, n = 2246, absolute risk difference of − 5.0%; 95% CI − 6.5% to − 3.6%; high certainty in evidence). Patients who received nirmatrelvir/ritonavir had fewer serious adverse events than placebo recipients (one RCT, n = 2246, absolute risk difference of − 4.9%; 95% CI − 6.5% to − 3.3%; high certainty in evidence).

*Recommendation 5*: We suggest using remdesivir in outpatients with mild COVID-19 (conditional recommendation, low certainty in evidence).

Summary of evidence: The review identified 430 references, and one RCT (PINETREE study) evaluating the effectiveness of remdesivir in the population of interest was included [17]. The trial tested intravenous remdesivir, 200 mg administered on day one, followed by 100 mg on days 2 and 3. Compared with placebo, remdesivir reduced hospitalisation (one RCT, n = 562, absolute risk difference of − 4.4%; 95% CI − 7.5% to − 1.3%; moderate certainty in evidence). Serious adverse events were more frequently observed in the remdesivir group (one RCT, n = 562, absolute risk difference of − 4.8%; 95% CI − 8.0% to − 1.5%; moderate certainty in evidence). No deaths occurred during the study follow-up.

*Recommendation 6*: we recommend against using hydroxychloroquine or chloroquine in outpatients with mild COVID-19 (strong recommendation, moderate certainty in evidence).

Summary of evidence: the review identified 783 references and six RCTs (ALBERTA HOPE COVID-19 study, COPE—COALITION COVID-19 Brazil V study, Mitjà et al.; Omrani et al.; Skipper et al. and TOGETHER study) evaluating the effectiveness of hydroxychloroquine or chloroquine in the population of interest were included [18–23]. The largest trial (COPE—COALITION COVID-19 Brazil V study) tested the administration of 400 mg of hydroxychloroquine twice daily on day 1, followed by 400 mg once daily after that, for seven days [18]. As compared with placebo, hydroxychloroquine or chloroquine did not significantly reduce mortality (six RCTs, n = 2981, absolute risk difference of 0.0%; 95% CI − 1.0% to 0.0%; moderate certainty in evidence) or hospitalisation (six RCTs, n = 2981, absolute risk difference of − 2.0%; 95% CI, − 3.0% to 0.0%; moderate certainty in evidence). No impact was observed on severe adverse events (five RCTs, n = 2558, absolute risk difference of 0.0%; 95% CI − 2.0% to 1.0%; moderate certainty in evidence).

*Recommendation 7*: we recommend against using ivermectin in outpatients with mild COVID-19 (strong recommendation, low certainty in evidence).

Summary of evidence: the review identified 168 references, and three RCTs (ACTIV-6 study, López-Medina et al. and TOGETHER study) evaluating the effectiveness of ivermectin in the population of interest were included [24–26]. All trials assessed efficacy (death and hospitalisation) and safety outcomes (adverse events).

Two trials tested ivermectin 400 μg/kg of body weight administered once daily for three days [25, 26], and one trial tested ivermectin 300 μg/kg administered once daily for five days [24]. As compared with placebo, ivermectin did not reduce mortality (three RCTs, n = 3425, absolute risk difference of 0.0%; 95% CI − 1.0% to 1.0%; moderate certainty in evidence) or hospitalisation (three RCTs, n = 3425, absolute risk difference of − 2.0%; 95% CI − 3.0% to 0.0%; moderate certainty in evidence). Ivermectin did not increase the incidence of serious adverse events (three RCTs, n = 3425, absolute risk difference of 0.0%; 95% CI − 2.0% to 1.0%; moderate certainty in evidence).

### Hospitalised patients with COVID-19

*Recommendation 8*: we suggest using remdesivir in hospitalised patients with severe COVID-19 (conditional recommendation, low certainty in evidence).

Summary of evidence: the review identified 430 references and eight RCTs (Abd-Elsalam et al. ACTT-1 study, CATCO study, DISCOVERY study, Mahajan et al. SIMPLE-Moderate study, Wuhan-Hubei study, and WHO Solidarity study) evaluating the effectiveness of remdesivir in the population of interest were included [27–34]. A 200 mg dose of remdesivir was administered on day 1, followed by 100 mg once daily for 4 to 9 days. As compared with the standard of care, remdesivir significantly reduced progression to invasive mechanical ventilation (eight RCTs, n = 11,857, absolute risk difference of − 3%; 95% CI − 5% to − 1%; low certainty in evidence) and showed a non-significant reduction in mortality (eight RCTs, n = 12,608, absolute risk difference of − 1%; 95% CI − 3% to 0%; moderate certainty in evidence). In addition, Remdesivir did not increase the incidence of serious adverse events (five RCTs, n = 2715, absolute risk difference of − 3%; 95% CI − 8% to 2%; very low certainty in evidence).

*Recommendation 9*: we suggest using baricitinib in hospitalised patients with severe COVID-19 (conditional recommendation, moderate certainty in evidence).

Summary of evidence: the review identified 75 references, and one RCT (COV-BARRIER study) evaluating the effectiveness of baricitinib in the population of interest was included [35, 36]. The COV-BARRIER study assessed the administration of baricitinib 4 mg once daily (oral or nasogastric tube) for 14 days or until hospital discharge. As compared with the standard of care, baricitinib significantly reduced mortality (one RCT, n = 1525, absolute risk difference of − 5.0%; 95% CI − 8.1% to − 1.9%; moderate certainty in evidence). In addition, Baricitinib did not increase the incidence of serious adverse events (one RCT, n = 1525, absolute risk difference of − 2.5%; 95% CI − 6.2% to 1.1%; low certainty in evidence).

*Recommendation 10*: we suggest using tocilizumab in hospitalised patients with severe COVID-19 (conditional recommendation, moderate certainty in evidence).

Summary of evidence: the review identified 358 references, and 14 RCTs evaluating the effectiveness of tocilizumab in the population of interest were included [37–48]. The intervention used in the most prominent trial (RECOVERY) consisted of the intravenous infusion of a single tocilizumab dose of 800 mg if weight > 90 kg, 600 mg if weight > 65 and ≤ 90 kg, 400 mg if weight > 40 and ≤ 65 kg, or 8 mg/kg if weight ≤ 40 kg, and a second dose could be administered 12 to 24 h later if, in the opinion of the clinician, the patient's condition had not improved [37]. As compared with the standard of care, tocilizumab significantly reduced mortality (14 RCTs, n = 7866, absolute risk difference of − 3.0%; 95% CI − 5.0% to − 1.0%; moderate certainty in evidence) and progression to mechanical ventilation (seven RCTs, n = 6866, absolute risk difference of − 2.0%; 95% CI − 4.% to − 1.0%; moderate certainty in evidence). Tocilizumab did not increase the incidence of serious adverse events (11 RCTs, n = 2489, absolute risk difference of − 1.0%; 95% CI − 5.0% to 2.0%; moderate certainty in evidence).

## Discussion

This joint SBI-API evidence-based guideline was developed by a panel of experts based on a comprehensive systematic review with meta-analysis of RCTs focused on ascertaining the efficacy of therapies in the prevention and treatment of COVID-19. The guideline provides ten recommendations that include tixagevimab + cilgavimab in the prophylaxis of COVID-19, tixagevimab + cilgavimab, molnupiravir, nirmatrelvir + ritonavir, and remdesivir in the treatment of outpatients, and remdesivir, baricitinib, and tocilizumab in the treatment of hospitalised patients with severe COVID-19. In addition, the use of hydroxychloroquine or chloroquine and ivermectin was discouraged.

Among COVID-19 confirmed infections, an appropriate treatment is key. Additionally, biomarkers would help in monitoring progression and evolution of disease, including C-reactive protein and procalcitonin, among others [49].

Some clinical treatments have been recommended in previous guidelines. Monoclonal antibodies (e.g. tixagevimab + cilgavimab), direct-acting antiviral agents (e.g. remdesivir), corticosteroids (e.g. dexamethasone), interleukin-6 antagonists (e.g. tocilizumab) and Janus kinase inhibitors (e.g., baricitinib) have been evaluated in guidelines for the treatment of patients with COVID-19 after RCT results became available indicating their benefit in specific populations [50, 51]. In Brazil, two guidelines were published for pharmacological treatment in outpatients and hospitalised patients. The Brazilian guidelines for the treatment of outpatients with suspected or confirmed COVID-19 provide ten recommendations, most of which advice against the use of the candidate technologies, contraindicating the clinical treatment of COVID-19 with anticoagulants, azithromycin, budesonide, colchicine, corticosteroids, hydroxychloroquine/chloroquine alone or combined with azithromycin, ivermectin, nitazoxanide, or convalescent plasma [52]. Using monoclonal antibodies in outpatients was impossible because of their uncertain benefits and high costs, with availability and implementation limitations [52]. The Brazilian guidelines for the pharmacological treatment of hospitalised patients with COVID-19 provide 16 recommendations that include treatment with corticosteroids in patients receiving supplemental oxygen and the use of prophylactic doses of anticoagulants for venous thromboembolism. In contrast, several medications were not recommended for this population, and even some studies on them, have been retracted [53].

Close to the scope of the current guideline, the renowned Infectious Diseases Society of America (IDSA) published guidelines on treating and managing patients with COVID-19 with 32 recommendations for prophylaxis in both outpatient and inpatient settings [54]. The IDSA guidelines apply to all patients with COVID-19, but some recommendations may differ based on disease severity [54]. The WHO definitions of disease severity for COVID-19 are as follows: (a) critical COVID-19—defined by the criteria for acute respiratory distress syndrome, sepsis, septic shock, or other conditions that would generally require the provision of life-sustaining therapies such as mechanical ventilation (invasive or noninvasive) or vasopressor therapy; (b) severe COVID-19—defined by oxygen saturation < 90% on room air, severe pneumonia, or signs of severe respiratory distress; and (c) non-severe COVID-19—defined as an absence of any criteria for severe or critical COVID-19 [54].

Although substantial progress has been made in COVID-19 treatment, some gaps remain. These include recommendations for treatment given the new SARS-CoV-2 variants of concern [55], as recruitment preceded the emergence of the omicron variant in most trials. The Pan-American Health Organization (PAHO) published an update on the emergence of omicron sublineages from SARS-CoV-2 recombination events [56]. In 2021, the omicron variant was introduced in the Americas and rapidly replaced delta and other lineages across the region and globally, becoming prevalent in all countries in the Americas since early 2022 [57–59]. The new emerging omicron sublineages carry additional S protein mutations, including BA.4.6 (with increasing incidence worldwide), BA.2.75.2 (with a growing incidence in India), BJ.1 (with increasing incidence mainly in India and Bangladesh), and BQ.1.1 (with a growing incidence in the USA and Europe) [55, 60]. On January 2023, the XBB.1.5 will be responsible for 61.3% of cases in the USA, following BQ.1.1 for 21.8% [61].

Emerging omicron sublineages resist some clinically used monoclonal antibodies, but preliminary data indicate complete resistance to XBB.1.5, BA.1.1 and BQ.1.1 to all monoclonal antibodies [55, 60, 62]. Therefore, in regions where this sublineage is spreading, patients may not respond well to clinical treatment with monoclonal antibodies alone, suggesting additional treatment options (e.g., nirmatrelvir/ritonavir or molnupiravir) should be considered for patients at high risk [60].

According to the FDA, over 90% of circulating variants are unlikely to be susceptible to tixagevimab-cilgavimab [62]. In this context, some organisations and societies remarked on neutralising antibodies. For example, on January 13, the IDSA added a remark to the neutralising antibodies for pre-exposure prophylaxis with tixagevimab/cilgavimab (Evusheld) recommendation due to resistance in the USA [54]. Also, the recommendation of neutralising antibodies for post-exposure prophylaxis with casirivimab/imdevimab was removed and replaced with a statement mentioning in vitro resistance to circulating strains in the USA [54].

Omicron sublineages BQ.1.1 and XBB1.5 can lead to a high volume of hospitalisations, which can strain healthcare systems and maintain a substantial number of deaths. That underscores the importance of preparing care units, specifically, hospital surge capacity and the ability to adequately staff health care systems and equip the health professionals who will care for these patients. In addition to vaccination, following recommended prevention strategies is essential to prevent poor outcomes such as infections, severe illness, and death from COVID-19 [8].

Deciding on the best practice has been challenging, given the rapid generation of large amounts of data and sometimes conflicting clinical results [51]. Nevertheless, despite limited evidence, this guideline recommends using agents in the prophylaxis and treatment of outpatients and hospitalised patients, considering an application context encompassing the Americas. Thus, the scope of this guideline proved to be comprehensive by answering the main clinical questions based on a robust method such as GRADE.

Although not discussed at this guideline nor the expert panel, we agree on the use of corticosteroids, as strong recommendation in favour, in patients with severe and critical COVID-19 confirmed infection, alone or combined with IL-6 receptor blockers or baricitinib, as recommended by WHO [63, 64].

The current guideline addresses pharmacological treatment in three different COVID-19 management scenarios contextualised in clinical practice in countries in the Americas. Further RCTs will be needed to update current recommendations as the pandemic still progresses in 2023.

## Conclusions

Since the beginning of the COVID-19 pandemic, studies have been conducted to provide the evidence necessary to formulate recommendations. This guideline presents a set of drugs that have proven effective in the prophylaxis and treatment of COVID-19 following the principles of evidence-based medicine, emphasising the strong recommendation for the use of nirmatrelvir/ritonavir in outpatients. Evidence has shown the lack of benefit of hydroxychloroquine and ivermectin, contraindicating their use in both outpatient and inpatient settings. It is strongly advised that these recommendations be adopted in the Americas to optimise the use of health resources and reduce the heterogeneity of procedures, as well as to reduce the progression to long COVID-19 [65].

### Supplementary Information

**Additional file 1: **The additional material includes information on the construction process of these guidelines, as well as the results of the synthesis and evaluation of the evidence. **Table S1.** Search strategies for systematic reviews. **Table S2.** Disclosure of financial interests for panel members involved on recommendations. **Table S3.** Should Tixagevimab + Cilgavimab treatment be recommended for pre-exposure prophylaxis in people at high risk of developing severe COVID-19?. **Table S4.** Should monoclonal antibody (Tixagevimab + Cilgavimab) treatment be recommended for outpatients with mild COVID-19?^a^. **Table S5.** Should molnupiravir treatment be recommended for outpatients with mild COVID-19 without risk factors for severe disease?. **Table S6.** Should molnupiravir treatment be recommended for outpatients with mild COVID-19 with risk factors for severe disease?. **Table S7.** Should Nirmatrelvir/ ritonarir treatment be recommended for outpatients with mild COVID-19?. **Table S8.** Should Remdesivir treatment be recommend for outpatients with mild COVID-19?. **Table S9.** Should Hidroxychloroquine treatment be recommended for outpatients with mild COVID-19?. **Table S10.** Should Ivermectin treatment be recommended for outpatients with mild COVID-19?. **Table S11.** Should Remdesivir treatment be recommended for hospitalized patients with severe COVID-19?. **Table S12.** Should Baracitinib treatment be recommended for hospitalized patients with severe COVID-19?. **Table S13.** Should Baracitinib treatment vs. dexamethasone be recommended for hospitalized patients with severe COVID-19?. **Table S14.** Should Tocilizumab treatment be recommended for hospitalized patients with severe COVID-19?. **Table S15.** Evidence to decision framework for recommending Tixagevimab + Cilgavimab treatment of pre-exposure prophylaxis in people at high risk of developing COVID-19. **Table S16.** Evidence to decision framework for recommending Tixagevimab + Cilgavimab treatment in outpatients with mild COVID-19. **Table S17.** Evidence to decision framework for recommending Molnupiravir treatment in outpatients with mild COVID-19. **Table S18.** Evidence to decision framework for recommending Nirmatrevir/Ritonavir treatment in outpatients with mild COVID-19. **Table S19.** Evidence to decision framework for recommending Remdesivir treatment in outpatients with mild COVID-19. **Table S20.** Evidence to decision framework for recommending Hidroxychloroquine or Chloroquine treatment in outpatients with mild COVID-19. **Table S21**. Evidence to decision framework for recommending Ivermectin treatment in outpatients with mild COVID-19. **Table S22.** Evidence to decision framework for recommending Remdesivir treatment in hospitalized patients with severe COVID-19. **Table S23.** Evidence to decision framework for recommending Baricitinib treatment in hospitalized patients with severe COVID-19. **Table S24.** Evidence to decision framework for recommending Tocilizumab treatment in hospitalized patients with severe COVID-19. **Figure S1.** Flow chart of study selection of Tixagevimab and Cilgavimab in Covid-19 pre-exposure prophylaxis. **Figure S2.** Flow chart of study selection of monoclonal antibody in outpatients with mild COVID-19. **Figure S3.** Flow chart of study selection of Nirmatrelvir plus Ritonavir in outpatients with mild COVID-19. **Figure S4.** Flow chart of study selection of Molnupiravir in outpatients with mild COVID-19. **Figure S5.** Flow chart of study selection of Remdesivir in outpatients with mild COVID-19. **Figure S6.** Flow chart of study selection of Hidroxychloroquine and Chloroquine in outpatients mild COVID-19. **Figure S7.** Flow chart of study selection of Ivermectin in outpatients mild COVID-19. **Figure S8.** Flow chart of study selection of Rendesivir in hospitalized patients with severe COVID-19. **Figure S9.** Flow chart of study selection of Baracitinib in hospitalized patients with severe COVID-19. **Figure S10.** Flow chart of study selection of Tocilizumab in hospitalized patients with severe COVID-19. **Figure S11.** Effect of Molnupiravir compared to control on mortality of outpatients with mild COVID-19. **Figure S12.** Effect of Molnupiravir compared to control on hospitalization of outpatients with mild COVID-19. **Figure S13.** Effect of Molnupiravir compared to control on serious adverse events in outpatients with mild COVID-19. **Figure S14.** Effect of Hidroxychloroquine and Chloroquine compared to control on mortality of outpatients with mild COVID-19. **Figure S15.** Effect of Hidroxychloroquine and Chloroquine compared to control on hospitalization of outpatients with mild COVID-19. **Figure S16.** Effect of Hidroxychloroquine and Chloroquine compared to control on serious adverse events in outpatients with mild COVID-19. **Figure S17.** Effect of Ivermectin compared to control on hospitalization of outpatients with mild COVID-19. **Figure S18.** Effect of Ivermectin compared to control on serious adverse events in outpatients with mild COVID-19. **Figure S19.** Effect of Remdesivir compared to control on mortality of hospitalized patients with severe COVID-19. **Figure S20.** Effect of Remdesivir compared to control on mechanical ventilation of hospitalized patients with severe COVID-19. **Figure S21.** Effect of Remdesivir compared to control on serious adverse events in hospitalized patients with severe COVID-19. **Figure S22.** Effect of Tocilizumab compared to control on mortality in hospitalized patients with severe COVID-19. **Figure S23.** Effect of Tocilizumab compared to control on mechanical ventilation in hospitalized patients with severe COVID-19. **Figure S24.** Effect of Tocilizumab compared to control on serious adverse events in hospitalized patients with severe COVID-19. **Figure S25.** Risk of bias assessment for the study of Tixagevimab + Cilgavimab in COVID-19 pre-exposure prophylaxis. **Figure S26.** Risk of bias assessment for the study of Tixagevimab + Cilgavimab in outpatients with mild COVID-19. **Figure S27.** Risk of bias assessment for the studies of Molnupiravir in outpatients with mild COVID-19. **Figure S28.** Risk of bias assessment for the study of Remdesivir in outpatients with mild COVID-19. **Figure S29.** Risk of bias assessment for the study of Nirmatrelvir plus Ritonavir in outpatients with mild COVID-19. **Figure S30.** Risk of bias assessment for the studies of Hidroxychloroquine and Chloroquine in outpatients with mild COVID-19. **Figure S31.** Risk of bias assessment for the studies of Ivermectin in outpatients with mild COVID-19. **Figure S32.** Risk of bias assessment for the study of Baricitinib in hospitalized patients with severe COVID-19. **Figure S33.** Risk of bias assessment for the studies of Tocilizumab in hospitalized patients with severe COVID-19.

## Data Availability

The dataset supporting the conclusions of this article is within the manuscript and its Additional file 1.

## Abbreviations

API: Pan-American Association of Infectious Diseases
CI: Confidence interval
COVID-19: Coronavirus disease 2019
EtD: Evidence to decision
GRADE: Grading of Recommendations Assessment, Development, and Evaluation
IDSA: Infectious Diseases Society of America
PAHO: Pan-American Health Organization
PICO: Patients, intervention, comparator, and outcome
PRISMA: Preferred Reporting Items for Systematic Reviews and Meta-Analyses
RCT: Randomised controlled trial
SARS-CoV-2: Severe acute respiratory syndrome coronavirus 2
SBI: Brazilian Society of Infectious Diseases
WHO: World Health Organization

## References

[1] Therapeutics and COVID-19: Living guideline. 2023. https://www.who.int/publications/i/item/WHO-2019-nCoV-therapeutics-2022.5. Accessed 31 Jan 2023.

[2] Al-Tawfiq JA, Rodriguez-Morales AJ (2020). Super-spreading events and contribution to transmission of MERS, SARS, and SARS-CoV-2 (COVID-19). J Hosp Infect.

[3] COVID-19 Dashboard. https://coronavirus.jhu.edu/map.html. Accessed 31 Jan 2023.

[4] WHO Coronavirus (COVID-19) Dashboard https://covid19.who.int/table. Accessed 31 Jan 2023.

[5] Iqbal Yatoo M, Hamid Z, Parray OR, Wani AH, Ul Haq A, Saxena A, Patel SK, Pathak M, Tiwari R, Malik YS (2020). COVID-19—recent advancements in identifying novel vaccine candidates and current status of upcoming SARS-CoV-2 vaccines. Hum Vaccin Immunother.

[6] Bergeri I, Whelan MG, Ware H, Subissi L, Nardone A, Lewis HC, Li Z, Ma X, Valenciano M, Cheng B (2022). Global SARS-CoV-2 seroprevalence from January 2020 to April 2022: a systematic review and meta-analysis of standardized population-based studies. PLoS Med.

[7] Solante R, Alvarez-Moreno C, Burhan E, Chariyalertsak S, Chiu NC, Chuenkitmongkol S, Dung DV, Hwang KP, Ortiz Ibarra J, Kiertiburanakul S (2023). Expert review of global real-world data on COVID-19 vaccine booster effectiveness and safety during the omicron-dominant phase of the pandemic. Expert Rev Vaccines.

[8] Iuliano AD, Brunkard JM, Boehmer TK, Peterson E, Adjei S, Binder AM, Cobb S, Graff P, Hidalgo P, Panaggio MJ (2022). Trends in disease severity and health care utilization during the early omicron variant period compared with previous SARS-CoV-2 high transmission periods—United States, December 2020–January 2022. MMWR Morb Mortal Wkly Rep.

[9] Andrews J, Guyatt G, Oxman AD, Alderson P, Dahm P, Falck-Ytter Y, Nasser M, Meerpohl J, Post PN, Kunz R (2013). GRADE guidelines: 14. Going from evidence to recommendations: the significance and presentation of recommendations. J Clin Epidemiol.

[10] Balshem H, Helfand M, Schünemann HJ, Oxman AD, Kunz R, Brozek J, Vist GE, Falck-Ytter Y, Meerpohl J, Norris S (2011). GRADE guidelines: 3. Rating the quality of evidence. J Clin Epidemiol.

[11] Handbook for grading the quality of evidence and the strength of recommendations using the GRADE approach. https://gdt.gradepro.org/app/handbook/handbook.html. Accessed 1 Aug 2023.

[12] Levin MJ, Ustianowski A, De Wit S, Launay O, Avila M, Templeton A, Yuan Y, Seegobin S, Ellery A, Levinson DJ (2022). Intramuscular AZD7442 (Tixagevimab–Cilgavimab) for prevention of Covid-19. N Engl J Med.

[13] Montgomery H, Hobbs FDR, Padilla F, Arbetter D, Templeton A, Seegobin S, Kim K, Campos JAS, Arends RH, Brodek BH (2022). Efficacy and safety of intramuscular administration of tixagevimab-cilgavimab for early outpatient treatment of COVID-19 (TACKLE): a phase 3, randomised, double-blind, placebo-controlled trial. Lancet Respir Med.

[14] Tippabhotla SKL, Lahiri S, Raju DR, Kandi C, Naga PV (2022). Efficacy and safety of molnupiravir for the treatment of non-hospitalized adults with mild COVID-19: a randomized, open-label, parallel-group phase 3 trial. SSRN.

[15] Jayk Bernal A, Da Silva MMG, Musungaie DB, Kovalchuk E, Gonzalez A, Delos Reyes V, Martín-Quirós A, Caraco Y, Williams-Diaz A, Brown ML (2022). Molnupiravir for oral treatment of Covid-19 in nonhospitalized patients. N Engl J Med.

[16] Hammond J, Leister-Tebbe H, Gardner A, Abreu P, Bao W, Wisemandle W, Baniecki M, Hendrick VM, Damle B, Simón-Campos A (2022). Oral nirmatrelvir for high-risk, nonhospitalized adults with Covid-19. N Engl J Med.

[17] Gottlieb RL, Vaca CE, Paredes R, Mera J, Webb BJ, Perez G, Oguchi G, Ryan P, Nielsen BU, Brown M (2022). Early remdesivir to prevent progression to severe Covid-19 in outpatients. N Engl J Med.

[18] Avezum Á, Oliveira GBF, Oliveira H, Lucchetta RC, Pereira VFA, Dabarian AL, Ricardo D, Vieira O, Silva DV, Kormann APM, Tognon AP (2022). Hydroxychloroquine versus placebo in the treatment of non-hospitalised patients with COVID-19 (COPE—Coalition V): a double-blind, multicentre, randomised, controlled trial. Lancet Reg Health Am.

[19] Mitjà O, Corbacho-Monné M, Ubals M, Tebé C, Peñafiel J, Tobias A, Ballana E, Alemany A, Riera-Martí N, Pérez CA (2021). Hydroxychloroquine for early treatment of adults with mild coronavirus disease 2019: a randomized, controlled trial. Clin Infect Dis.

[20] Reis G, Moreira Silva EADS, Medeiros Silva DC, Thabane L, Singh G, Park JJH, Forrest JI, Harari O, Dos Santos CVQ, De Almeida APFG (2021). Effect of early treatment with hydroxychloroquine or lopinavir and ritonavir on risk of hospitalization among patients with COVID-19. JAMA Netw Open.

[21] Schwartz I, Boesen ME, Cerchiaro G, Doram C, Edwards BD, Ganesh A, Greenfield J, Jamieson S, Karnik V, Kenney C (2021). Assessing the efficacy and safety of hydroxychloroquine as outpatient treatment of COVID-19: a randomized controlled trial. CMAJ Open.

[22] Skipper CP, Pastick KA, Engen NW, Bangdiwala AS, Abassi M, Lofgren SM, Williams DA, Okafor EC, Pullen MF, Nicol MR (2020). Hydroxychloroquine in nonhospitalized adults with early COVID-19: a randomized trial. Ann Intern Med.

[23] Omrani AS, Pathan SA, Thomas SA, Harris TRE, Coyle PV, Thomas CE, Qureshi I, Bhutta ZA, Mawlawi NA, Kahlout RA (2020). Randomized double-blinded placebo-controlled trial of hydroxychloroquine with or without azithromycin for virologic cure of non-severe Covid-19. EClinicalMedicine.

[24] López-Medina E, López P, Hurtado IC, Dávalos DM, Ramirez O, Martínez E, Díazgranados JA, Oñate JM, Chavarriaga H, Herrera S (2021). Effect of ivermectin on time to resolution of symptoms among adults with mild COVID-19. JAMA.

[25] Naggie S, Boulware DR, Lindsell CJ, Stewart TG, Gentile N, Collins S, McCarthy MW, Jayaweera D, Castro M, Sulkowski M (2022). Effect of ivermectin vs placebo on time to sustained recovery in outpatients with mild to moderate COVID-19. JAMA.

[26] Reis G, Silva EASM, Silva DCM, Thabane L, Milagres AC, Ferreira TS, Dos Santos CVQ, Campos VHS, Nogueira AMR, De Almeida APFG (2022). Effect of early treatment with ivermectin among patients with Covid-19. N Engl J Med.

[27] WHO Solidarity Trial Consortium (2022). Remdesivir and three other drugs for hospitalised patients with COVID-19: final results of the WHO solidarity randomised trial and updated meta-analyses. Lancet.

[28] Abd-Elsalam S, Ahmed OA, Mansour NO, Abdelaziz DH, Salama M, Fouad MHA, Soliman S, Naguib AM, Hantera MS, Ibrahim IS (2021). Remdesivir efficacy in COVID-19 treatment: a randomized controlled trial. Am J Trop Med Hyg.

[29] Ali K, Azher T, Baqi M, Binnie A, Borgia S, Carrier FM, Cavayas YA, Chagnon N, Cheng MP, Conly J (2022). Remdesivir for the treatment of patients in hospital with COVID-19 in Canada: a randomized controlled trial. Can Med Assoc J.

[30] Beigel JH, Tomashek KM, Dodd LE, Mehta AK, Zingman BS, Kalil AC, Hohmann E, Chu HY, Luetkemeyer A, Kline S (2020). Remdesivir for the treatment of Covid-19—final report. N Engl J Med.

[31] Mahajan L, Singh AP (2021). Gifty: clinical outcomes of using remdesivir in patients with moderate to severe COVID-19—a prospective randomised study. Indian J Anaesth.

[32] Spinner CD, Gottlieb RL, Criner GJ, Arribas López JR, Cattelan AM, Soriano Viladomiu A, Ogbuagu O, Malhotra P, Mullane KM, Castagna A (2020). Effect of remdesivir vs standard care on clinical status at 11 days in patients with moderate COVID-19. JAMA.

[33] Wang Y, Zhang D, Du G, Du R, Zhao J, Jin Y, Fu S, Gao L, Cheng Z, Lu Q (2020). Remdesivir in adults with severe COVID-19: a randomised, double-blind, placebo-controlled, multicentre trial. The Lancet.

[34] Ader F, Bouscambert-Duchamp M, Hites M, Peiffer-Smadja N, Poissy J, Belhadi D, Diallo A, Lê M-P, Peytavin G, Staub T (2022). Remdesivir plus standard of care versus standard of care alone for the treatment of patients admitted to hospital with COVID-19 (DisCoVeRy): a phase 3, randomised, controlled, open-label trial. Lancet Infect Dis.

[35] Ely EW, Ramanan AV, Kartman CE, De Bono S, Liao R, Piruzeli MLB, Goldman JD, Saraiva JFK, Chakladar S, Marconi VC (2022). Efficacy and safety of baricitinib plus standard of care for the treatment of critically ill hospitalised adults with COVID-19 on invasive mechanical ventilation or extracorporeal membrane oxygenation: an exploratory, randomised, placebo-controlled trial. Lancet Respir Med.

[36] Marconi VC, Ramanan AV, De Bono S, Kartman CE, Krishnan V, Liao R, Piruzeli MLB, Goldman JD, Alatorre-Alexander J, De Cassia PR (2021). Efficacy and safety of baricitinib for the treatment of hospitalised adults with COVID-19 (COV-BARRIER): a randomised, double-blind, parallel-group, placebo-controlled phase 3 trial. Lancet Respir Med.

[37] RECOVERY Collaborative Group (2021). Tocilizumab in patients admitted to hospital with COVID-19 (RECOVERY): a randomised, controlled, open-label, platform trial. Lancet.

[38] Broman N, Feuth T, Vuorinen T, Valtonen M, Hohenthal U, Löyttyniemi E, Hirvioja T, Jalava-Karvinen P, Marttila H, Nordberg M (2022). Early administration of tocilizumab in hospitalized COVID-19 patients with elevated inflammatory markers; COVIDSTORM-a prospective, randomized, single-centre, open-label study. Clin Microbiol Infect.

[39] Hermine O, Mariette X, Porcher R, Djossou F, Nguyen Y, Arlet JB, Savale L, Diehl JL, Georgin-Lavialle S, Cadranel J (2022). Tocilizumab plus dexamethasone versus dexamethasone in patients with moderate-to-severe COVID-19 pneumonia: a randomised clinical trial from the CORIMUNO-19 study group. EClinicalMedicine.

[40] Hermine O, Mariette X, Tharaux PL, Resche-Rigon M, Porcher R, Ravaud P, CORIMUNO-19 Collaborative Group (2021). Effect of tocilizumab vs usual care in adults hospitalized With COVID-19 and moderate or severe pneumonia: a randomized clinical trial. JAMA Intern Med.

[41] Rosas IO, Bräu N, Waters M, Go R, Hunter BD, Bhagani S, Skiest D, Aziz MS, Cooper N, Douglas IS (2021). Tocilizumab in hospitalized patients with COVID-19 pneumonia. N Engl J Med.

[42] Salama C, Han J, Yau L, Reiss WG, Kramer B, Neidhart JD, Criner GJ, Kaplan-Lewis E, Baden R, Pandit L (2020). Tocilizumab in patients hospitalized with Covid-19 pneumonia. N Engl J Med.

[43] Salvarani C, Dolci G, Massari M, Merlo DF, Cavuto S, Savoldi L, Bruzzi P, Boni F, Braglia L, Turrà C (2021). Effect of tocilizumab vs standard care on clinical worsening in patients hospitalized with COVID-19 pneumonia: a randomized clinical trial. JAMA Intern Med.

[44] Stone JH, Frigault MJ, Serling-Boyd NJ, Fernandes AD, Harvey L, Foulkes AS, Horick NK, Healy BC, Shah R, Bensaci AM (2020). Efficacy of tocilizumab in patients hospitalized with Covid-19. N Engl J Med.

[45] Veiga VC, Prats JAGG, Farias DLC, Rosa RG, Dourado LK, Zampieri FG, Machado FR, Lopes RD, Berwanger O, Azevedo LCP (2021). Effect of tocilizumab on clinical outcomes at 15 days in patients with severe or critical coronavirus disease 2019: randomised controlled trial. BMJ.

[46] Declercq J, Van Damme KFA, De Leeuw E, Maes B, Bosteels C, Tavernier SJ, De Buyser S, Colman R, Hites M, Verschelden G (2021). Effect of anti-interleukin drugs in patients with COVID-19 and signs of cytokine release syndrome (COV-AID): a factorial, randomised, controlled trial. Lancet Respir Med.

[47] Soin AS, Kumar K, Choudhary NS, Sharma P, Mehta Y, Kataria S, Govil D, Deswal V, Chaudhry D, Singh PK (2021). Tocilizumab plus standard care versus standard care in patients in India with moderate to severe COVID-19-associated cytokine release syndrome (COVINTOC): an open-label, multicentre, randomised, controlled, phase 3 trial. Lancet Respir Med.

[48] Derde LPG (2021). Effectiveness of tocilizumab, sarilumab, and anakinra for critically ill patients with COVID-19 the REMAP-CAP COVID-19 immune modulation therapy domain randomized clinical trial.

[49] Camon AM, Alonso R, Muñoz FJ, Cardozo C, Bernal-Maurandi J, Albiach L, Agüero D, Marcos MA, Ambrosioni J, Bodro M (2022). C-reactive protein cut-off for early tocilizumab and dexamethasone prescription in hospitalized patients with COVID-19. Sci Rep.

[50] Singh M, De Wit E (2022). Antiviral agents for the treatment of COVID-19: progress and challenges. Cell Rep Med.

[51] Wohl DA, Espinueva AA, Dau L, Wang C-Y, Lachmann A, Bam RA, Rawal A, Chappell-Smith K, Rockstroh JK (2022). COVID-19 therapies for inpatients: a review and quality assessment of clinical guidelines. ERJ Open Res.

[52] Falavigna M, Belli KC, Barbosa AN, Zavascki AP, Nastri ACDSS, Santana CM, Stein C, Gräf DD, Cadegiani FA, Guimarães HP (2022). Brazilian guidelines for the treatment of outpatients with suspected or confirmed COVID-19. A joint guideline of the Brazilian Association of Emergency Medicine (ABRAMEDE), Brazilian Medical Association (AMB), Brazilian Society of Angiology and Vascular S. Br J Infect Dis.

[53] Falavigna M, Stein C, Amaral JLGD, Azevedo LCPD, Belli KC, Colpani V, Cunha CAD, Dal-Pizzol F, Dias MBS, Ferreira JC (2022). Diretrizes Brasileiras para o tratamento farmacológico de pacientes hospitalizados com COVID-19. Revista Brasileira de Terapia Intensiva.

[54] Bhimraj A MR, Shumaker AH, Baden L, Cheng VC, Edwards KM, Gallagher JC, Gandhi RT, Muller WJ, Nakamura MM, O'Horo JC, Shafer RW, Shoham S, Murad MH, Mustafa RA, Sultan S, Falck-Ytter Y. Infectious Diseases Society of America Guidelines on the treatment and management of patients with COVID-19. Version 10.1.1. Infectious Diseases Society of America. 2022.

[55] Farahat RA, Abdelaal A, Umar TP, El-Sakka AA, Benmelouka AY, Albakri K, Ali I, Al-Ahdal T, Abdelazeem B, Sah R (2022). The emergence of SARS-CoV-2 Omicron subvariants: current situation and future trends. Infez Med.

[56] Atualização sobre a emergência de sublinhagens de Ômicron de SARS- CoV-2 eventos de recombinação. https://www.paho.org/pt/documentos/atualizacao-sobre-emergencia-sublinhagens-omicron-sars-cov-2-eventos-recombinacao. Accessed 31 Jan 2023.

[57] WH Organization (2022). TAG-VE statement on omicron sublineages BQ.1 and XBB.

[58] Poudel S, Ishak A, Perez-Fernandez J, Garcia E, León-Figueroa DA, Romaní L, Bonilla-Aldana DK, Rodriguez-Morales AJ (2022). Highly mutated SARS-CoV-2 omicron variant sparks significant concern among global experts—what is known so far?. Travel Med Infect Dis.

[59] Nishiura H, Ito K, Anzai A, Kobayashi T, Piantham C, Rodríguez-Morales AJ (2021). Relative reproduction number of SARS-CoV-2 omicron (B.1.1.529) compared with delta variant in South Africa. J Clin Med.

[60] Arora P, Kempf A, Nehlmeier I, Schulz SR, Jäck H-M, Pöhlmann S, Hoffmann M (2022). Omicron sublineage BQ.1.1 resistance to monoclonal antibodies. Lancet Infect Dis.

[61] COVID data tracker—variant proportions https://covid.cdc.gov/covid-data-tracker/#variant-proportions Accessed 31 Jan 2023.

[62] FDA announces Evusheld is not currently authorized for emergency use in the U.S. https://www.fda.gov/drugs/drug-safety-and-availability/fda-announces-evusheld-not-currently-authorized-emergency-use-us Accessed 31 Jan 2023.

[63] Lamontagne F, Stegemann M, Agarwal A, Agoritsas T, Siemieniuk R, Rochwerg B, Bartoszko J, Askie L, Macdonald H, Al-Maslamani M, Amin W (2023). Update to living WHO guideline on drugs to prevent covid-19. BMJ.

[64] Siemieniuk RA, Bartoszko JJ, Zeraatkar D, Kum E, Qasim A, Martinez JPD, Izcovich A, Lamontagne F, Han MA, Agarwal A (2020). Drug treatments for covid-19: living systematic review and network meta-analysis. BMJ.

[65] Rodriguez-Morales AJ, Lopez-Echeverri MC, Perez-Raga MF, Quintero-Romero V, Valencia-Gallego V, Galindo-Herrera N, López-Alzate S, Sánchez-Vinasco JD, Gutiérrez-Vargas JJ, Mayta-Tristan P (2023). The global challenges of the long COVID-19 in adults and children. Travel Med Infect Dis.

