# Supplementary material for: Pan-American Guidelines for the treatment of SARS-CoV-2/COVID-19: a joint evidence-based guideline of the Brazilian Society of Infectious Diseases (SBI) and the Pan-American Association of Infectious Diseases (API)
Source: Ann Clin Microbiol Antimicrob. 2023 Aug 7;22:67. doi: 10.1186/s12941-023-00623-w (PMC10408214; doi:10.1186/s12941-023-00623-w)
Supplement: Supplementary file 1 — Additional file 1: The additional material includes information on the construction process of these guidelines, as well as the results of the synthesis and evaluation of the evidence. Table S1. Search strategies for systematic reviews. Table S2. Disclosure of financial interests for panel members involved on recommendations. Table S3. Should Tixagevimab + Cilgavimab treatment be recommended for pre-exposure prophylaxis in people at high risk of developing severe COVID-19?. Table S4. Should monoclonal antibody (Tixagevimab + Cilgavimab) treatment be recommended for outpatients with mild COVID-19?a. Table S5. Should molnupiravir treatment be recommended for outpatients with mild COVID-19 without risk factors for severe disease?. Table S6. Should molnupiravir treatment be recommended for outpatients with mild COVID-19 with risk factors for severe disease?. Table S7. Should Nirmatrelvir/ ritonarir treatment be recommended for outpatients with mild COVID-19?. Table S8. Should Remdesivir treatment be recommend for outpatients with mild COVID-19?. Table S9. Should Hidroxychloroquine treatment be recommended for outpatients with mild COVID-19?. Table S10. Should Ivermectin treatment be recommended for outpatients with mild COVID-19?. Table S11. Should Remdesivir treatment be recommended for hospitalized patients with severe COVID-19?. Table S12. Should Baracitinib treatment be recommended for hospitalized patients with severe COVID-19?. Table S13. Should Baracitinib treatment vs. dexamethasone be recommended for hospitalized patients with severe COVID-19?. Table S14. Should Tocilizumab treatment be recommended for hospitalized patients with severe COVID-19?. Table S15. Evidence to decision framework for recommending Tixagevimab + Cilgavimab treatment of pre-exposure prophylaxis in people at high risk of developing COVID-19. Table S16. Evidence to decision framework for recommending Tixagevimab + Cilgavimab treatment in outpatients with mild COVID-19. Table S17. Evidence to [file 12941_2023_623_MOESM1_ESM.docx]

**Additional material**

**List of Additional tables**

Additional Table S1. Search strategies for systematic reviews 5

Additional Table S2. Disclosure of interests for panel members involved on recommendations 6

Additional Table S3. Should Tixagevimab + Cilgavimab treatment be recommended for pre-exposure prophylaxis in people at high risk of developing severe COVID-19? 7

Additional Table S4. Should monoclonal antibody (Tixagevimab + Cilgavimab) treatment be recommended for outpatients with mild COVID-19? ^a^ 8

Additional Table S5. Should molnupiravir treatment be recommended for outpatients with mild COVID-19 without risk factors for severe disease? 9

Additional Table S6. Should molnupiravir treatment be recommended for outpatients with mild COVID-19 with risk factors for severe disease? 10

Additional Table S7. Should Nirmatrelvir/ ritonarir treatment be recommended for outpatients with mild COVID-19? 11

Additional Table S8. Should Remdesivir treatment be recommend for outpatients with mild COVID-19? 12

Additional Table S9. Should Hidroxychloroquine treatment be recommended for outpatients with mild COVID-19? 13

Additional Table S10. Should Ivermectin treatment be recommended for outpatients with mild COVID-19? 14

Additional Table S11. Should Remdesivir treatment be recommended for hospitalized patients with severe COVID-19? 15

Additional Table S12. Should Baracitinib treatment be recommended for hospitalized patients with severe COVID-19? 16

Additional Table S13.Should Baracitinib treatment vs. dexamethasone be recommended for hospitalized patients with severe COVID-19? 17

Additional Table S14. Should Tocilizumab treatment be recommended for hospitalized patients with severe COVID-19? 18

Additional Table S15. Evidence to decision framework for recommending Tixagevimab + Cilgavimab treatment of pre-exposure prophylaxis in people at high risk of developing COVID-19 19

Additional Table S16. Evidence to decision framework for recommending Tixagevimab + Cilgavimab treatment in outpatients with mild COVID-19 20

Additional Table S17. Evidence to decision framework for recommending Molnupiravir treatment in outpatients with mild COVID-19 21

Additional Table S18. Evidence to decision framework for recommending Nirmatrevir/Ritonavir treatment in outpatients with mild COVID-19 22

Additional Table S19. Evidence to decision framework for recommending Remdesivir treatment in outpatients with mild COVID-19 23

Additional Table S20. Evidence to decision framework for recommending Hidroxychloroquine or Chloroquine treatment in outpatients with mild COVID-19 24

Additional Table S21. Evidence to decision framework for recommending Ivermectin treatment in outpatients with mild COVID-19 25

Additional Table S22. Evidence to decision framework for recommending Remdesivir treatment in hospitalized patients with severe COVID-19 26

Additional Table S23. Evidence to decision framework for recommending Baricitinib treatment in hospitalized patients with severe COVID-19 27

Additional Table S24. Evidence to decision framework for recommending Tocilizumab treatment in hospitalized patients with severe COVID-19 28

**List of Additional figures**

Additional Figure S1. Flow chart of study selection of Tixagevimab and Cilgavimab in Covid-19 pre-exposure prophylaxis 29

Additional Figure S2. Flow chart of study selection of monoclonal antibody in outpatients with mild COVID-19 30

Additional Figure S3. Flow chart of study selection of Nirmatrelvir plus Ritonavir in outpatients with mild COVID-19 31

Additional Figure S4. Flow chart of study selection of Molnupiravir in outpatients with mild COVID-19 32

Additional Figure S5. Flow chart of study selection of Remdesivir in outpatients with mild COVID-19 33

Additional Figure S6. Flow chart of study selection of Hidroxychloroquine and Chloroquine in outpatients mild COVID-19 34

Additional Figure S7. Flow chart of study selection of Ivermectin in outpatients mild COVID-19 35

Additional Figure S8. Flow chart of study selection of Rendesivir in hospitalized patients with severe COVID-19 36

Additional Figure S9. Flow chart of study selection of Baracitinib in hospitalized patients with severe COVID-19 37

Additional Figure S10. Flow chart of study selection of Tocilizumab in hospitalized patients with severe COVID-19 38

Additional Figure S11. Effect of Molnupiravir compared to control on mortality of outpatients with mild COVID-19 39

Additional Figure S12. Effect of Molnupiravir compared to control on hospitalization of outpatients with mild COVID-19 39

Additional Figure S13. Effect of Molnupiravir compared to control on serious adverse events in outpatients with mild COVID-19 39

Additional Figure S14. Effect of Hidroxychloroquine and Chloroquine compared to control on mortality of outpatients with mild COVID-19 39

Additional Figure S15. Effect of Hidroxychloroquine and Chloroquine compared to control on hospitalization of outpatients with mild COVID-19 40

Additional Figure S16. Effect of Hidroxychloroquine and Chloroquine compared to control on serious adverse events in outpatients with mild COVID-19 40

Additional Figure S17. Effect of Ivermectin compared to control on hospitalization of outpatients with mild COVID-19 40

Additional Figure S18. Effect of Ivermectin compared to control on serious adverse events in outpatients with mild COVID-19 41

Additional Figure S19. Effect of Remdesivir compared to control on mortality of hospitalized patients with severe COVID-19 41

Additional Figure S20. Effect of Remdesivir compared to control on mechanical ventilation of hospitalized patients with severe COVID-19 41

Additional Figure S21. Effect of Remdesivir compared to control on serious adverse events in hospitalized patients with severe COVID-19 42

Additional Figure S22. Effect of Tocilizumab compared to control on mortality in hospitalized patients with severe COVID-19 42

Additional Figure S23. Effect of Tocilizumab compared to control on mechanical ventilation in hospitalized patients with severe COVID-19 42

Additional Figure S24. Effect of Tocilizumab compared to control on serious adverse events in hospitalized patients with severe COVID-19 43

Additional Figure S25. Risk of bias assessment for the study of Tixagevimab + Cilgavimab in COVID-19 pre-exposure prophylaxis 44

Additional Figure S26. Risk of bias assessment for the study of Tixagevimab + Cilgavimab in outpatients with mild COVID-19 44

Additional Figure S27. Risk of bias assessment for the studies of Molnupiravir in outpatients with mild COVID-19 44

Additional Figure S28. Risk of bias assessment for the study of Remdesivir in outpatients with mild COVID-19 45

Additional Figure S29. Risk of bias assessment for the study of Nirmatrelvir plus Ritonavir in outpatients with mild COVID-19 45

Additional Figure S30. Risk of bias assessment for the studies of Hidroxychloroquine and Chloroquine in outpatients with mild COVID-19 45

Additional Figure S31. Risk of bias assessment for the studies of Ivermectin in outpatients with mild COVID-19 46

Additional Figure S32. Risk of bias assessment for the study of Baricitinib in hospitalized patients with severe COVID-19 46

Additional Figure S33. Risk of bias assessment for the studies of Tocilizumab in hospitalized patients with severe COVID-19 46

Additional table S1. Search strategies for systematic reviews

| **Question** | **Search Strategy*** |
| --- | --- |
| Question 1 | (AZD7442 OR Tixagevimab OR Cilgavimab) AND (COVID-19 OR COVID OR coronavirus OR SARS-CoV-2) AND Random* |
| Question 2 | (Casirivimab OR Imdevimab OR Bamlanivimab OR Etesivimab OR Sotrovimab OR Regdanvimab OR Tixagevimab OR Cilgavimab OR Bebtelovimab  OR Monoclonal Antibodies OR Monoclonal Antibody) AND (COVID OR COV OR CORONAVIRUS OR SARS) AND Random* |
| Question 3 | (Molnupiravir) AND (COVID OR COV OR CORONAVIRUS OR SARS) AND Random* |
| Question 4 | (Nirmatrelvir) AND (COVID OR COV OR CORONAVIRUS OR SARS) AND Random* |
| Question 5 | (Remdesivir) AND (COVID OR COV OR CORONAVIRUS OR SARS) AND Random* |
| Question 6 | (IVERMECTIN) AND (COVID OR COV OR CORONAVIRUS OR SARS) AND Random* |
| Question 7 | (Chloroquine OR Chlorochin OR Hydroxychloroquine OR Oxychloroquine OR Hydroxychlorochin) AND (COVID OR COV OR CORONAVIRUS OR SARS) AND Random* |
| Question 8 | (Remdesivir) AND (COVID OR COV OR CORONAVIRUS OR SARS) AND Random* |
| Question 9 | (sars cov 2 OR sars cov 2 OR covid OR covid 19 OR covid 19 OR COV OR coronavirus OR coronavirus OR coronaviruses OR SARS) AND (baricitinib) AND random* |
| Question 10 | (sars cov 2 OR sars cov 2 OR covid OR covid 19 OR covid 19 OR COV OR coronavirus OR coronavirus OR coronaviruses OR SARS) AND (tocilizumab) AND random* |

*Search update: July 6^th^, 2022.

Additional table S2. Disclosure of financial interests for panel members involved on recommendations

| **Name** | **Disclosure of interests** | **Questions with potential financial conflict of interest^a^** |
| --- | --- | --- |
| Alberto Chebabo | - | 1, 2 (tixagebimab + cilgavimab); |
| Alexandre Naime Barbosa | - | 1, 2 (tixagebimab + cilgavimab); 3 (molnupiravir); 5, 8 (remdesivir) |
| Alfonso Javier Rodriguez-Morales | No direct financial interests | Not applicable |
| Carlos Starling | - | 1, 2 (tixagebimab + cilgavimab) |
| Clevy Pérez | No direct financial interests | Not applicable |
| Clóvis Arns Cunha | - | 1, 2 (tixagebimab + cilgavimab); 3 (molnupiravir); 5 (remdesivir) |
| David de Luna | No direct financial interests | Not applicable |
| Estevão Portela Nunes | - | 5, 8 (remdesivir) |
| Gabriela Zambrano | No direct financial interests | Not applicable |
| Juliana Carvalho Ferreira | No direct financial interests | Not applicable |
| Júlio Croda | - | 3 (molnupiravir); 4 (Nirmatrevir/ritonavir) |
| Monica Maria Gomes da Silva | - | 3 (molnupiravir); |
| Monica Thormann | No direct financial interests | Not applicable |
| Sérgio Cimerman | - | 1, 2 (tixagebimab + cilgavimab); 3 (molnupiravir); 5, 8 (remdesivir) |
| Suzana Tanni | No direct financial interests | Not applicable |

^a^ Members with a direct financial conflict of interest related to a given intervention did not vote for the related questions

Additional table S3. Should Tixagevimab + Cilgavimab treatment be recommended for pre-exposure prophylaxis in people at high risk of developing severe COVID-19?

| **Certainty assessment** | | | | | | | **№ of patients** | | **Effect** | | **Certainty** | **Importance** |
| --- | --- | --- | --- | --- | --- | --- | --- | --- | --- | --- | --- | --- |
| **№ of studies** | **Study design** | **Risk of bias** | **Inconsistency** | **Indirectness** | **Imprecision** | **Other considerations** | **Tixagevimab + cilgavimab** | **Placebo** | **Relative (95% CI)** | **Absolute (95% CI)** |  |  |
| **Symptomatic COVID-19 episode** | | | | | | | | | | | | |
| 1 | randomised trials | very serious^a^ | not serious | not serious | not serious | none | 20/3461 (0.6%) | 44/1736 (2.5%) | not estimable | - | ⨁⨁◯◯ Low | CRITICAL |
| **Adverse event with death** | | | | | | | | | | | | |
| 1 | randomised trials | very serious^a^ | not serious | not serious | serious^b^ | none | 4/3461 (0.1%) | 4/1736 (0.2%) | not estimable | - | ⨁◯◯◯ Very low | CRTICAL |
| **Serious adverse event** | | | | | | | | | | | | |
| 1 | randomised trials | very serious^a^ | not serious | not serious | serious^b^ | none | 50/3461 (1.4%) | 23/1736 (1.3%) | not estimable | - | ⨁◯◯◯ Very low | IMPORTANT |

**CI:** Confidence interval

#### **Explanations**

a. Follow-up loss greater than 20%.

b. Optimal Information Size not met.

Additional table S4. Should monoclonal antibody (Tixagevimab + Cilgavimab) treatment be recommended for outpatients with mild COVID-19? ^a^

| **Certainty assessment** | | | | | | | **№ de pacientes** | | **Efeito** | | **Certainty** | **Importance** |
| --- | --- | --- | --- | --- | --- | --- | --- | --- | --- | --- | --- | --- |
| **№ of studies** | **Study design** | **Risk of bias** | **Inconsistency** | **Indirectness** | **Imprecision** | **Other considerations** | **Tixagevimab + cilgavimab** | **Placebo** | **Relative (95% CI)** | **Absolut (95% CI)** |  |  |
| **Mortality** | | | | | | | | | | | | |
| 1 | randomised trials | not serious | not serious | not serious | serious^b^ | none | 6/456 (1.3%) | 6/454 (1.3%) | not estimable | **0 fewer per 100** (from 1 fewer to 1 more) | ⨁⨁⨁◯ Moderate | CRTICAL |
| **Hospitalization** | | | | | | | | | | | | |
| 1 | randomised trials | not serious | not serious | not serious | not serious | none | 17/456 (3.7%) | 40/454 (8.8%) | not estimable | **5 fewer per 100** (from 8 fewer to 2 fewer) | ⨁⨁⨁⨁ High | IMPORTANT |
| **Serious adverse event** | | | | | | | | | | | | |
| 1 | randomised trials | not serious | not serious | not serious | not serious | none | 22/456 (4.8%) | 30/454 (6.6%) | not estimable | **2 fewer per 100** (from 5 fewer to 1 more) | ⨁⨁⨁⨁ High | IMPORTANT |

**CI:** Confidence interval

#### Explanations

a. Due to the lack of effectiveness for the omicron variant, the panel chose not to make recommendations for Bamlanivimab, Casirivimab, Etesivimab, Imdevimab, Regdanvimab and Sotrovimab. For Bebtelovimab, no recommendation was made due to lack of evidence.

b. Optimal Information Size not met.

Additional table S5. Should molnupiravir treatment be recommended for outpatients with mild COVID-19 without risk factors for severe disease?

| **Certainty assessment** | | | | | | | **№ of patients** | | **Effect** | | **Certainty** | **Importance** |
| --- | --- | --- | --- | --- | --- | --- | --- | --- | --- | --- | --- | --- |
| **№ of studies** | **Study design** | **Risk of bias** | **Inconsistency** | **Indirectness** | **Imprecision** | **Other considerations** | **Molnupiravir** | **Placebo** | **Relative (95% CI)** | **Absolut (95% CI)** |  |  |
| **Mortality** | | | | | | | | | | | | |
| 2 | randomised trials | serious^a, b, c^ | not serious | not serious | not serious | none | 0/610 (0.0%) | 0/610 (0.0%) | not estimable | **10 more per 1.000** (from 10 fewer to 20 more) | ⨁⨁⨁◯ Moderate | CRITICAL |
| **Hospitalization** | | | | | | | | | | | | |
| 2 | randomised trials | serious^a, b, c^ | not serious | not serious | not serious | none | 7/610 (1.2%) | 13/610 (2.1%) | not estimable | **10 more per 1.000** (from 0 fewer to 30 more) | ⨁⨁⨁◯ Moderate | CRITICAL |
| **Serious Adverse Events** | | | | | | | | | | | | |
| 2 | randomised trials | serious^a, b, c^ | not serious | not serious | not serious | none | 78/610 (12.8%) | 81/610 (13.3%) | not estimable | **0 per 1.000** (from 40 fewer to 30 more) | ⨁⨁⨁◯ Moderate | IMPORTANT |

**CI:** confidence interval

#### Explanations

a. No blinding.

b. Absence of blinding, analysis by ITT and sample calculation.

b. No sample size calculation.

Additional table S6. Should molnupiravir treatment be recommended for outpatients with mild COVID-19 with risk factors for severe disease?

| **Certainty assessment** | | | | | | | **№ of patients** | | **Effect** | | **Certainty** | **Importance** |
| --- | --- | --- | --- | --- | --- | --- | --- | --- | --- | --- | --- | --- |
| **№ of studies** | **Study design** | **Risk of bias** | **Inconsistency** | **Indirectness** | **Imprecision** | **Other considerations** | **Molnupiravir** | **Placebo** | **Relative (95% CI)** | **Absolut (95% CI)** |  |  |
| **Mortality** | | | | | | | | | | | | |
| 1 | randomised trial | not serious | not serious | not serious | not serious | none | 1/716 (0.1%) | 9/717 (1.3%) | not estimable | **10 more per 1.000** (from 20 fewer to 0 fewer) | ⨁⨁⨁⨁ High | CRITICAL |
| **Hospitalization** | | | | | | | | | | | | |
| 1 | randomised trial | not serious | not serious | not serious | not serious | none | 47/716 (6.6%) | 59/717 (8.2%) | not estimable | **20 more per 1.000** (from 40 fewer to 10 more) | ⨁⨁⨁⨁ High | CRITICAL |
| **Serious Adverse Events** | | | | | | | | | | | | |
| 1 | randomised trial | not serious | not serious | not serious | not serious | none | 49/716 (6.8%) | 67/717 (9.3%) | not estimable | **30 more per 1.000** (from 50 fewer to 0 more) | ⨁⨁⨁⨁ High | IMPORTANT |

**CI:** confidence interval

#### Explanations

Additional table S7. Should Nirmatrelvir/ ritonarir treatment be recommended for outpatients with mild COVID-19?

| **Certainty assessment** | | | | | | | **№ of patients** | | **Effect** | | **Certainty** | **Importance** |
| --- | --- | --- | --- | --- | --- | --- | --- | --- | --- | --- | --- | --- |
| **№ of studies** | **Study design** | **Risk of bias** | **Inconsistency** | **Indirectness** | **Imprecision** | **Other considerations** | **Nirmatrelvir + Ritonavir** | **Placebo** | **Relative (95% CI)** | **Absolut (95% CI)** |  |  |
| **Mortality** | | | | | | | | | | | | |
| 1 | randomised trials | not serious | not serious | not serious | serious^a^ | none | 0/1120 (0.0%) | 12/1126 (1.1%) | not estimable | - | ⨁⨁⨁◯ Moderate | CRITICAL |
| **Hospitalization** | | | | | | | | | | | | |
| 1 | randomised trials | not serious | not serious | not serious | not serious | none | 8/1120 (0.7%) | 65/1126 (5.8%) | not estimable | - | ⨁⨁⨁⨁ High | CRITICAL |
| **Adverse Events** | | | | | | | | | | | | |
| 1 | randomised trials | not serious | not serious | not serious | not serious | none | 18/1120 (1.6%) | 74/1126 (6.6%) | not estimable | - | ⨁⨁⨁⨁ High | IMPORTANT |

**CI:** Confidence interval

#### Explanations

a. Optimal Information Size not met.

Additional table S8. Should Remdesivir treatment be recommend for outpatients with mild COVID-19?

| **Certainty assessment** | | | | | | | **№ of patients** | | **Effect** | | **Certainty** | **Importance** |
| --- | --- | --- | --- | --- | --- | --- | --- | --- | --- | --- | --- | --- |
| **№ of studies** | **Study design** | **Risk of bias** | **Inconsistency** | **Indirectness** | **Imprecision** | **Other considerations** | **Remdesivir** | **Placebo** | **Relative (95% CI)** | **Absolut (95% CI)** |  |  |
| **Mortality** | | | | | | | | | | | | |
| 1 | randomised trials | serious^a^ | not serious | not serious | serious^b^ | none | 0/292 (0.0%) | 0/292 (0.0%) | not estimable | - | ⨁⨁◯◯ Low | CRITICAL |
| **Hospitalization** | | | | | | | | | | | | |
| 1 | randomised trials | serious^a^ | not serious | not serious | not serious | none | 5/292 (1.7%) | 18/292 (6.2%) | not estimable | - | ⨁⨁⨁◯ Moderate | CRITICAL |
| **Serious Adverse Events** | | | | | | | | | | | | |
| 1 | randomised trials | serious^a^ | not serious | not serious | not serious | none | 5/292 (1.7%) | 19/292 (6.5%) | not estimable | - | ⨁⨁⨁◯ Moderate | IMPORTANT |

**CI:** Confidence interval

#### Explanations

a. Early discontinuation of the study.

b. Optimal Information Size not met.

Additional Table S9. Should Hidroxychloroquine treatment be recommended for outpatients with mild COVID-19?

| **Certainty assessment** | | | | | | | **№ of patients** | | **Effect** | | **Certainty** | **Importance** |
| --- | --- | --- | --- | --- | --- | --- | --- | --- | --- | --- | --- | --- |
| **№ of studies** | **Study design** | **Risk of bias** | **Inconsistency** | **Indirectness** | **Imprecision** | **Other considerations** | **HCQ** | **Placebo** | **Relative (95% CI)** | **Absolut (95% CI)** |  |  |
| **Mortality** | | | | | | | | | | | | |
| 6 | randomised trials | serious^c^ | not serious | not serious | not serious | none | 6/1514 (0.4%) | 7/1467 (0.5%) | not estimable | **0 fewer per 1.000** (from 0 fewer to 10 more) | ⨁⨁⨁◯ Moderate | CRITICAL |
| **Hospitalization** | | | | | | | | | | | | |
| 6 | randomised trials | serious^a^ | not serious | not serious | not serious | none | 71/1514 (4.7%) | 93/1467 (6.3%) | not estimable | **20 more per 1.000** (from 0 fewer to 30 more) | ⨁⨁⨁◯ Moderate | CRITICAL |
| **Serious Adverse Events** | | | | | | | | | | | | |
| 5 | randomised trials | serious^b^ | not serious | not serious | not serious | none | 41/1302 (3.1%) | 45/1256 (3.6%) | not estimable | **0 fewer per 1.000** (from 10 fewer to 20 more) | ⨁⨁⨁◯ Moderate | IMPORTANT |

**CI:** Confidence interval; **HCQ:** Hidroxychloroquine

#### Explanations

a. Follow-up loss greater than 20%.

b. Absence of analysis by ITT.

c. Absence of blinding.

Additional Table S10. Should Ivermectin treatment be recommended for outpatients with mild COVID-19?

| **Certainty assessment** | | | | | | | **№ of patients** | | **Effect** | | **Certainty** | **Importance** |
| --- | --- | --- | --- | --- | --- | --- | --- | --- | --- | --- | --- | --- |
| **№ of studies** | **Study design** | **Risk of bias** | **Inconsistency** | **Indirectness** | **Imprecision** | **Other considerations** | **Ivermectin** | **Placebo** | **Relative (95% CI)** | **Absolut (95% CI)** |  |  |
| **Mortality** | | | | | | | | | | | | |
| 3 | randomised trials | serious^a^ | not serious | not serious | not serious | none | 22/1734 (1.3%) | 25/1691 (1.5%) | not estimable | **0 fewer per 1000** (from 10 fewer to 10 more) | ⨁⨁⨁◯ Moderate | CRITICAL |
| **Hospitalization** | | | | | | | | | | | | |
| 3 | randomised trials | serious^a^ | not serious | not serious | not serious | none | 110/1734 (6.3%) | 124/1691 (7.3%) | not estimable | **10 fewer per 1000** (from 10 fewer to 20 more) | ⨁⨁⨁◯ Moderate | CRITICAL |
| **Serious Adverse Event** | | | | | | | | | | | | |
| 3 | randomised trials | serious^a^ | not serious | not serious | not serious | none | 50/1734 (2.9%) | 53/1691 (3.1%) | not estimable | **0 fewer per 1000** (from 10 fewer to 10 more) | ⨁⨁⨁◯ Moderate | IMPORTANT |

**CI:** Confidence interval

#### Explanations

a. Limitation on sample size calculation, ITT analysis and unclear risk of bias.

Additional Table S11. Should Remdesivir treatment be recommended for hospitalized patients with severe COVID-19?

| **Certainty assessment** | | | | | | | **№ of patients** | | **Effect** | | **Certainty** | **Importance** |
| --- | --- | --- | --- | --- | --- | --- | --- | --- | --- | --- | --- | --- |
| **№ of studies** | **Study design** | **Risk of bias** | **Inconsistency** | **Indirectness** | **Imprecision** | **Other considerations** | **Remdesivir** | **Placebo** | **Relative (95% CI)** | **Absolut (95% CI)** |  |  |
| **Mortality** | | | | | | | | | | | | |
| 8 | randomised trials | serious^a^ | not serious | not serious | not serious | none | 863/6451 (13.4%) | 934/6157 (15.2%) | not estimable | **10 more per 1.000** (from 0 fewer to 30 more) | ⨁⨁⨁◯ Moderate | CRITICAL |
| **Mechanical Ventilation or ECMO** | | | | | | | | | | | | |
| 8 | randomised trials | serious^a^ | serious^b^ | not serious | not serious | none | 677/6069 (11.2%) | 822/5788 (14.2%) | not estimable | **30 more per 1.000** (from 10 more to 50 more) | ⨁⨁◯◯ Low | CRITICAL |
| **Serious Adverse Events** | | | | | | | | | | | | |
| 5 | randomised trials | serious^a^ | very serious^c^ | not serious | serious^d^ | none | 297/1399 (21.2%) | 331/1316 (25.2%) | not estimable | **30 more per 1.000** (from 20 fewer to 80 more) | ⨁◯◯◯ Very low | IMPORTANT |

**CI:** confidence interval

#### Explanations

a. Absence of blinding.

b. Heterogeneity 50% - 75%.

c. Heterogeneity > 75%.

d. Large 95% CI.

Additional Table S12. Should Baracitinib treatment be recommended for hospitalized patients with severe COVID-19?

| **Certainty assessment** | | | | | | | **№ of patients** | | **Effect** | | **Certainty** | **Importance** |
| --- | --- | --- | --- | --- | --- | --- | --- | --- | --- | --- | --- | --- |
| **№ of studies** | **Study design** | **Risk of bias** | **Inconsistency** | **Indirectness** | **Imprecision** | **Other considerations** | **Baricitinib** | **Placebo** | **Relative (95% CI)** | **Absolute (95% CI)** |  |  |
| **Mortality** | | | | | | | | | | | | |
| 1 | randomised trials | serious^a^ | not serious | not serious | not serious | none | 62/764 (8.1%) | 100/761 (13.1%) | not estimable | **5 fewer per 100** (from 8 fewer to 2 fewer) | ⨁⨁⨁◯ Moderate | CRITICAL |
| **Serious Adverse Events** | | | | | | | | | | | | |
| 1 | randomised trials | serious^a^ | not serious | not serious | serious^b^ | none | 111/764 (14.5%) | 130/761 (17.1%) | not estimable | **3 fewer per 100** (from 6 fewer to 1 more) | ⨁⨁◯◯ Low | IMPORTANT |

**CI:** confidence interval

#### Explanations

a. Follow-up loss greater than 20%.

b. Large 95% CI.

Additional Table S13.Should Baracitinib treatment vs. dexamethasone be recommended for hospitalized patients with severe COVID-19?

| **Certainty assessment** | | | | | | | **№ of patients** | | **Effect** | | **Certainty** | **Importance** |
| --- | --- | --- | --- | --- | --- | --- | --- | --- | --- | --- | --- | --- |
| **№ of studies** | **Study design** | **Risk of bias** | **Inconsistency** | **Indirectness** | **Imprecision** | **Other considerations** | **Baricitinib** | **Dexamethasone** | **Relative (95% CI)** | **Absolute (95% CI)** |  |  |
| **Mortality** | | | | | | | | | | | | |
| 1 | randomised trials | serious^a^ | not serious | not serious | serious^b^ | none | 27/516 (5.2%) | 30/494 (6.1%) | not estimable | **1 fewer per 100** (from 4 fewer to 2 more) | ⨁⨁◯◯ Low | CRITICAL |
| **Mechanical Ventilation or ECMO** | | | | | | | | | | | | |
| 1 | randomised trials | serious^a^ | not serious | not serious | serious^b^ | none | 57/516 (11.0%) | 50/494 (10.1%) | not estimable | **1 fewer per 100** (from 3 fewer to 5 more) | ⨁⨁◯◯ Low | CRITICAL |
| **Serious Adverse Events** | | | | | | | | | | | | |
| 1 | randomised trials | serious^a^ | not serious | not serious | serious^b^ | none | 95/516 (18.4%) | 94/494 (19.0%) | not estimable | **1 fewer per 100** (from 5 fewer to 4 more) | ⨁⨁◯◯ Low | IMPORTANT |

**CI:** confidence interval

#### Explanations

a. Follow-up loss greater than 20%.

b. Large 95% CI.

Additional Table S14. Should Tocilizumab treatment be recommended for hospitalized patients with severe COVID-19?

| **Certainty assessment** | | | | | | | **№ of patients** | | **Effect** | | **Certainty** | **Importance** |
| --- | --- | --- | --- | --- | --- | --- | --- | --- | --- | --- | --- | --- |
| **№ of studies** | **Study design** | **Risk of bias** | **Inconsistency** | **Indirectness** | **Imprecision** | **Other considerations** | **Tocilizumab** | **Placebo** | **Relative (95% CI)** | **Absolut (95% CI)** |  |  |
| **Mortality** | | | | | | | | | | | | |
| 14 | randomised trials | serious^a^ | not serious | not serious | not serious | none | 1089/4365 (24.9%) | 986/3501 (28.2%) | not estimable | **1 fewer per 1000** (from 10 fewer to 50 fewer) | ⨁⨁⨁◯ Moderate | CRITICAL |
| **Mechanical Ventilation** | | | | | | | | | | | | |
| 7 | randomised trials | serious^a^ | not serious | not serious | not serious | none | 389/3849 (10.1%) | 282/3017 (9.3%) | not estimable | **20** fewer **per 1000** (from 10 fewer to 40 fewer) | ⨁⨁⨁◯ Moderate | CRITICAL |
| **Adverse Events** | | | | | | | | | | | | |
| 11 | randomised trials | serious^a^ | not serious | not serious | not serious | none | 301/1436 (21.0%) | 227/1053 (21.6%) | not estimable | **10 more per 1000** (from 20 fewer to 50 more) | ⨁⨁⨁◯ Moderate | IMPORTANT |

**CI:** confidence interval

#### Explanations

a. Absence of blinding.

Additional Table 15. Evidence to decision framework for recommending Tixagevimab + Cilgavimab treatment of pre-exposure prophylaxis in people at high risk of developing COVID-19

| 1) Should Tixagevimab + Cilgavimab treatment be recommended for pre-exposure prophylaxis in people at high risk of developing severe COVID-19? | | |
| --- | --- | --- |
| **Domain** | **Question** | **Judgement** |
| Problem | Is the problem a priority? | ☐ No  ☐ Probably no  ☐ Probably yes  ☐ Yes   - Varies   ☐ Don’t know |
| Desirable effects | How substantial are the desirable anticipated effects? | ☐ Trivial  ☐ Small  ☐ Moderate   - Large   ☐ Varies  ☐ Don’t know |
| Undesirable effects | How substantial are the undesirable anticipated effects? | - Trivial   ☐ Small  ☐ Moderate  ☐ Large  ☐ Varies  ☐ Don’t know |
| Certainty of evidence | What is the overall certainty of the evidence of effects? | - Very low   ☐ Low  ☐ Moderate  ☐ High  ☐ No included studies |
| Balance of effects | Does the balance between desirable and undesirable effects favor the intervention or the comparison? | ☐ Favors the comparison  ☐ Probably favors the comparison ☐ Does not favor either the intervention or the comparison   - Probably favors the intervention   ☐ Favors the intervention  ☐ Varies  ☐ Don't know |
| Feasibility | Is the intervention feasible to implement? | ☐ No  ☐ Probably no   - Probably yes   ☐ Yes  ☐ Varies  ☐ Don’t know |
| Recommendation | We suggest using Tixagevimab + Cilgavimab for prophylaxis in people at high risk of developing severe COVID-19 (conditional recommendation, very low certainty in evidence). | |

Additional Table S16. Evidence to decision framework for recommending Tixagevimab + Cilgavimab treatment in outpatients with mild COVID-19

| Should Tixagevimab + Cilgavimab treatment be recommended for outpatients with mild COVID-19? | | |
| --- | --- | --- |
| **Domain** | **Question** | **Judgement** |
| Problem | Is the problem a priority? | ☐ No  ☐ Probably no  ☐ Probably yes   - Yes   ☐ Varies  ☐ Don’t know |
| Desirable effects | How substantial are the desirable anticipated effects? | ☐ Trivial  ☐ Pequena   - Moderada   ☐ Grande  ☐ Varia  ☐ Desconhecido |
| Undesirable effects | How substantial are the undesirable anticipated effects? | ☐ Trivial   - Small   ☐ Moderate  ☐ Large  ☐ Varies  ☐ Don’t know |
| Certainty of evidence | What is the overall certainty of the evidence of effects? | ☐ Very low  ☐ Low   - Moderate   ☐ High  ☐ No included studies |
| Balance of effects | Does the balance between desirable and undesirable effects favor the intervention or the comparison? | ☐ Favors the comparison  ☐ Probably favors the comparison ☐ Does not favor either the intervention or the comparison   - Probably favors the intervention   ☐ Favors the intervention  ☐ Varies  ☐ Don't know |
| Feasibility | Is the intervention feasible to implement? | ☐ No  ☐ Probably no   - Probably yes   ☐ Yes  ☐ Varies  ☐ Don’t know |
| Recommendation | We suggest using tixagevimab + cilgavimab for prophylaxis in outpatients with mild COVID-19 (conditional recommendation, moderate certainty in evidence). | |

Additional Table S17. Evidence to decision framework for recommending Molnupiravir treatment in outpatients with mild COVID-19

| Should Molnupiravir treatment be recommended for outpatients with mild COVID-19? | | |
| --- | --- | --- |
| **Domain** | **Question** | **Opções de resposta** |
| Problem | Is the problem a priority? | ☐ No  ☐ Probably no  ☐ Probably yes  ☐ Yes   - Varies   ☐ Don’t know |
| Desirable effects | How substantial are the desirable anticipated effects? | ☐ Trivial   - Small   ☐ Moderate  ☐ Large  ☐ Varies  ☐ Don’t know |
| Undesirable effects | How substantial are the undesirable anticipated effects? | - Trivial   ☐ Small  ☐ Moderate  ☐ Large  ☐ Varies  ☐ Don’t know |
| Certainty of evidence | What is the overall certainty of the evidence of effects? | - Very low   ☐ Low  ☐ Moderate  ☐ High  ☐ No included studies |
| Balance of effects | Does the balance between desirable and undesirable effects favor the intervention or the comparison? | ☐ Favors the comparison  ☐ Probably favors the comparison ☐ Does not favor either the intervention or the comparison   - Probably favors the intervention   ☐ Favors the intervention  ☐ Varies  ☐ Don't know |
| Feasibility | Is the intervention feasible to implement? | ☐ No  ☐ Probably no   - Probably yes   ☐ Yes  ☐ Varies  ☐ Don’t know |
| Recommendation | We suggest using Molnupiravir in outpatients with mild COVID-19 (conditional recommendation, very low certainty in evidence). | |

Additional Table S18. Evidence to decision framework for recommending Nirmatrevir/Ritonavir treatment in outpatients with mild COVID-19

| Should Nirmatrelvir/ritonavir treatment be recommended for outpatients with mild COVID-19? | | |
| --- | --- | --- |
| **Domain** | **Question** | **Judgement** |
| Problem | Is the problem a priority? | ☐ No  ☐ Probably no   - Probably yes   ☐ Yes  ☐ Varies  ☐ Don’t know |
| Desirable effects | How substantial are the desirable anticipated effects? | ☐ Trivial  ☐ Small  ☐ Moderate   - Large   ☐ Varies  ☐ Don’t know |
| Undesirable effects | How substantial are the undesirable anticipated effects? | ☐ Trivial   - Small   ☐ Moderate  ☐ Large  ☐ Varies  ☐ Don’t know |
| Certainty of evidence | What is the overall certainty of the evidence of effects? | ☐ Very low  ☐ Low   - Moderate   ☐ High |
| Balance of effects | Does the balance between desirable and undesirable effects favor the intervention or the comparison? | ☐ Favors the comparison  ☐ Probably favors the comparison ☐ Does not favor either the intervention or the comparison  ☐ Probably favors the intervention   - Favors the intervention   ☐ Varies  ☐ Don't know |
| Feasibility | Is the intervention feasible to implement? | ☐ No  ☐ Probably no   - Probably yes   ☐ Yes  ☐ Varies  ☐ Don’t know |
| Recommendation | We recommend using Nirmatrelvir/Ritonavir in outpatients with mild COVID-19 (strong recommendation, moderate certainty in evidence). | |

Additional Table S19. Evidence to decision framework for recommending Remdesivir treatment in outpatients with mild COVID-19

| Should Remdesivir treatment be recommend for outpatients with mild COVID-19? | | |
| --- | --- | --- |
| **Domain** | **Question** | **Judgement** |
| Problem | Is the problem a priority? | ☐ No  ☐ Probably no   - Probably yes   ☐ Yes  ☐ Varies  ☐ Don’t know |
| Desirable effects | How substantial are the desirable anticipated effects? | ☐ Trivial  ☐ Small   - Moderate   ☐ Large  ☐ Varies  ☐ Don’t know |
| Undesirable effects | How substantial are the undesirable anticipated effects? | - Trivial   ☐ Small  ☐ Moderate  ☐ Large  ☐ Varies  ☐ Don’t know |
| Certainty of evidence | What is the overall certainty of the evidence of effects? | ☐ Very low   - Low   ☐ Moderate  ☐ High  ☐ No included studies |
| Balance of effects | Does the balance between desirable and undesirable effects favor the intervention or the comparison? | ☐ Favors the comparison  ☐ Probably favors the comparison ☐ Does not favor either the intervention or the comparison   - Probably favors the intervention   ☐ Favors the intervention  ☐ Varies  ☐ Don't know |
| Feasibility | Is the intervention feasible to implement? | ☐ No  ☐ Probably no  ☐ Probably yes  ☐ Yes   - Varies   ☐ Don’t know |
| Recommendation | We suggest using Remdesivir in outpatients with mild COVID-19 (conditional recommendation, low certainty in evidence). | |

Additional Table S20. Evidence to decision framework for recommending Hidroxychloroquine or Chloroquine treatment in outpatients with mild COVID-19

| Should Hidroxychloroquine or Chloroquine treatment be recommended for outpatients with mild COVID-19? | | |
| --- | --- | --- |
| **Domain** | **Question** | **Judgement** |
| Problem | Is the problem a priority? | ☐ No  ☐ Probably no   - Probably yes   ☐ Yes  ☐ Varies  ☐ Don’t know |
| Desirable effects | How substantial are the desirable anticipated effects? | - Trivial   ☐ Small  ☐ Moderate  ☐ Large  ☐ Varies  ☐ Don’t know |
| Undesirable effects | How substantial are the undesirable anticipated effects? | ☐ Trivial   - Small   ☐ Moderate  ☐ Large  ☐ Varies  ☐ Don’t know |
| Certainty of evidence | What is the overall certainty of the evidence of effects? | ☐ Very low  ☐ Low   - Moderate   ☐ High  ☐ No included studies |
| Balance of effects | Does the balance between desirable and undesirable effects favor the intervention or the comparison? | ☐ Favors the comparison   - Probably favors the comparison   ☐ Does not favor either the intervention or the comparison  ☐ Probably favors the intervention  ☐ Favors the intervention  ☐ Varies  ☐ Don't know |
| Feasibility | Is the intervention feasible to implement? | ☐ No  ☐ Probably no  ☐ Probably yes   - Yes   ☐ Varies  ☐ Don’t know |
| Recommendation | We recommend against using Hidroxychloroquine or Chloroquine in outpatients with mild COVID-19 (strong recommendation, moderate certainty in evidence). | |

Additional Table 21. Evidence to decision framework for recommending Ivermectin treatment in outpatients with mild COVID-19

| Should Ivermectin treatment be recommended for outpatients with mild COVID-19? | | |
| --- | --- | --- |
| **Domain** | **Question** | **Judgement** |
| Problem | Is the problem a priority? | ☐ No  ☐ Probably no   - Probably yes   ☐ Yes  ☐ Varies  ☐ Don’t know |
| Desirable effects | How substantial are the desirable anticipated effects? | - Trivial   ☐ Small  ☐ Moderate  ☐ Large  ☐ Varies  ☐ Don’t know |
| Undesirable effects | How substantial are the undesirable anticipated effects? | ☐ Trivial   - Small   ☐ Moderate  ☐ Large  ☐ Varies  ☐ Don’t know |
| Certainty of evidence | What is the overall certainty of the evidence of effects? | ☐ Very low  ☐ Low   - Moderate   ☐ High  ☐ No included studies |
| Balance of effects | Does the balance between desirable and undesirable effects favor the intervention or the comparison? | ☐ Favors the comparison   - Probably favors the comparison   ☐ Does not favor either the intervention or the comparison  ☐ Probably favors the intervention  ☐ Favors the intervention  ☐ Varies  ☐ Don't know |
| Feasibility | Is the intervention feasible to implement? | ☐ No  ☐ Probably no  ☐ Probably yes   - Yes   ☐ Varies  ☐ Don’t know |
| Recommendation | We recommend against using Ivermectin in outpatients with mild COVID-19 (strong recommendation, moderate certainty in evidence) | |

Additional Table S22. Evidence to decision framework for recommending Remdesivir treatment in hospitalized patients with severe COVID-19

| Should Remdesivir treatment be recommended for hospitalized patients with severe COVID-19? | | |
| --- | --- | --- |
| **Domain** | **Question** | **Judgement** |
| Problem | Is the problem a priority? | ☐ No  ☐ Probably no  ☐ Probably yes   - Yes   ☐ Varies  ☐ Don’t know |
| Desirable effects | How substantial are the desirable anticipated effects? | ☐ Trivial  ☐ Small   - Moderate   ☐ Large  ☐ Varies  ☐ Don’t know |
| Undesirable effects | How substantial are the undesirable anticipated effects? | ☐ Trivial   - Small   ☐ Moderate  ☐ Large  ☐ Varies  ☐ Don’t know |
| Certainty of evidence | What is the overall certainty of the evidence of effects? | ☐ Very low   - Low   ☐ Moderate  ☐ High  ☐ No included studies |
| Balance of effects | Does the balance between desirable and undesirable effects favor the intervention or the comparison? | ☐ Favors the comparison  ☐ Probably favors the comparison ☐ Does not favor either the intervention or the comparison   - Probably favors the intervention   ☐ Favors the intervention  ☐ Varies  ☐ Don't know |
| Feasibility | Is the intervention feasible to implement? | ☐ No  ☐ Probably no   - Probably yes   ☐ Yes  ☐ Varies  ☐ Don’t know |
| Recommendation | We suggest using Remdesivir in hospitalized patients with severe COVID-19 (conditional recommendation, low certainty in evidence). | |

Additional Table S23. Evidence to decision framework for recommending Baricitinib treatment in hospitalized patients with severe COVID-19

| Should Baracitinib treatment be recommended for hospitalized patients with severe COVID-19? | | |
| --- | --- | --- |
| **Domain** | **Question** | **Judgement** |
| Problem | Is the problem a priority? | ☐ No  ☐ Probably no  ☐ Probably yes   - Yes   ☐ Varies  ☐ Don’t know |
| Desirable effects | How substantial are the desirable anticipated effects? | ☐ Trivial  ☐ Small   - Moderate   ☐ Large  ☐ Varies  ☐ Don’t know |
| Undesirable effects | How substantial are the undesirable anticipated effects? | ☐ Trivial   - Small   ☐ Moderate  ☐ Large  ☐ Varies  ☐ Don’t know |
| Certainty of evidence | What is the overall certainty of the evidence of effects? | ☐ Trivial  ☐ Small   - Moderate   ☐ Large  ☐ Varies  ☐ Don’t know |
| Balance of effects | Does the balance between desirable and undesirable effects favor the intervention or the comparison? | ☐ Favors the comparison  ☐ Probably favors the comparison ☐ Does not favor either the intervention or the comparison   - Probably favors the intervention   ☐ Favors the intervention  ☐ Varies  ☐ Don't know |
| Feasibility | Is the intervention feasible to implement? | ☐ No  ☐ Probably no   - Probably yes   ☐ Yes  ☐ Varies  ☐ Don’t know |
| Recommendation | We suggest using Baricitinib in hospitalized patients with severe COVID-19 (conditional recommendation, moderate certainty in evidence). | |

Additional Table S24. Evidence to decision framework for recommending Tocilizumab treatment in hospitalized patients with severe COVID-19

| Should Tocilizumab treatment be recommended for hospitalized patients with severe COVID-19? | | |
| --- | --- | --- |
| **Domain** | **Question** | **Judgement** |
| Problem | Is the problem a priority? | ☐ No  ☐ Probably no  ☐ Probably yes   - Yes   ☐ Varies  ☐ Don’t know |
| Desirable effects | How substantial are the desirable anticipated effects? | ☐ Trivial  ☐ Small   - Moderate   ☐ Large  ☐ Varies  ☐ Don’t know |
| Undesirable effects | How substantial are the undesirable anticipated effects? | ☐ Trivial   - Small   ☐ Moderate  ☐ Large  ☐ Varies  ☐ Don’t know |
| Certainty of evidence | What is the overall certainty of the evidence of effects? | ☐ Trivial  ☐ Small   - Moderate   ☐ Large  ☐ Varies  ☐ Don’t know |
| Balance of effects | Does the balance between desirable and undesirable effects favor the intervention or the comparison? | ☐ Favors the comparison  ☐ Probably favors the comparison ☐ Does not favor either the intervention or the comparison   - Probably favors the intervention   ☐ Favors the intervention  ☐ Varies  ☐ Don't know |
| Feasibility | Is the intervention feasible to implement? | ☐ No  ☐ Probably no   - Probably yes   ☐ Yes  ☐ Varies  ☐ Don’t know |
| Recommendation | We suggest using Tocilizumab in hospitalized patients with severe COVID-19 (conditional recommendation, moderate certainty in evidence). | |

Records identified through databases searching (n = 13)

Medline: n = 2, Embase: n = 2, Clinical Trials: n = 2 and Scholar: n = 7

Manual search (n = 0)

Identification

Screening

Record after duplicates removed (n = 13)

**Records excluded (n = 12)**

Treatment (n = 2)

Duplicate (n = 4)

Interview (n = 1)

Unrelated (n = 1)

Comment (n = 1)

Letter to the editor (n = 1)

Case series (n = 1)

Cohort (n = 1)

Full-text article assessed for eligibility (n = 13)

Eligibility

**Study included in qualitative synthesis (n = 1)**

**Study included in quantitative synthesis (n = 1)**

Included

Additional figure 1. Flow chart of study selection of Tixagevimab and Cilgavimab in Covid-19 pre-exposure prophylaxis

Records identified through databases searching (n = 53)

Medline: n = 26, Embase: n = 15, Clinical Trials: n = 3 and Scholar: n = 9

Manual search (n = 0)

Identification

**Record excluded bases on title and/or abstract**

(n = 0)

Screening

Records after duplicates removed (n = 53)

**Records excluded (n = 52)**

Inpatients (n = 1)

Prevention (n = 11)

Opinionated evidence (n = 13)

Exploratory (n = 1)

Unrelated with doubt (n = 3)

Letter/editorial (n = 5)

In vitro/animal model (n = 4)

Review (n = 6)

Case series (n: 5)

No results (n = 3)

Full-text article assessed for eligibility (n = 53)

Eligibility

**Study included in qualitative synthesis (n = 1)**

**Study included in quantitative synthesis (n = 1)**

Included

Additional figure 2. Flow chart of study selection of monoclonal antibody in outpatients with mild COVID-19

Records identified through databases searching (n = 19)

Medline: n = 11, Embase: n = 3, Clinical Trials: n = 4 and Scholar: n = 1

Manual search (n = 0)

Identification

Screening

**Record excluded bases on title and/or abstract**

(n = 9)

Records screened after duplicates removed (n = 19)

**Record excluded (n = 9)**

RCT phase 1 (n = 3)

Review (n = 1)

Observational (n = 1)

Recruiting (n = 4)

Full-text article assessed for eligibility (n = 10)

Eligibility

**Study included in qualitative synthesis (n = 1)**

**Study included in quantitative synthesis (n = 1)**

Included

Additional figure 3. Flow chart of study selection of Nirmatrelvir plus Ritonavir in outpatients with mild COVID-19

Records identified through databases searching (n = 27)

Medline: n =16, Embase: n =3, Clinical Trials: n = 5 and Scholar: n = 2

Manual search (n = 1)

Identification

Screening

**Record excluded bases on title and/or abstract**

(n = 10)

Records screened after duplicates removed (n = 27)

**Record excluded (n = 14)**

RCT phase 1 (n = 3)

RCT phase 2 (n = 4)

Review (n = 2)

Preprint (n = 1) Pharmacokinetics outcome (n = 1)

Recruiting (n = 2)

Protocols (n = 2)

Full-text article assessed for eligibility (n = 17)

Eligibility

**Study included in qualitative synthesis (n = 2)**

**Study included in quantitative synthesis (n = 2)**

Included

Additional figure 4. Flow chart of study selection of Molnupiravir in outpatients with mild COVID-19

Records identified through databases searching (n = 430)

Medline: n = 329, Embase: n = 51, Clinical Trials: n = 12 and Scholar: n = 38

Manual search (n = 0)

Identification

Screening

**Record excluded bases on title and/or abstract** (n = 378)

Records screened after duplicates removed (n = 426)

**Record excluded (n = 47)**

Inpatient (n = 10)

Association (n = 6)

Cohort (n = 8)

Non-randomized (n = 1)

Comment (n = 4)

Another drug (n = 2)

Abstract (n = 9)

No results (n = 2)

Post hoc (n = 2)

Model (n = 2)

Review (n = 1)

Full-text article assessed for eligibility (n = 48)

Eligibility

**Study included in qualitative synthesis (n = 1)**

**Study included in quantitative synthesis (n = 1)**

Included

Additional figure 5. Flow chart of study selection of Remdesivir in outpatients with mild COVID-19

Records identified through databases searching (n = 783)

Manual search (n = 0)

Identification

Screening

**Record excluded bases on title and/or abstract** (n = 719)

Records screened after duplicates removed (n = 783)

**Record excluded (n = 58)**

Inpatient (n = 14)

Another outcome (n = 9)

Comment (n = 1)

Meta-analysis (n = 1)

RCT phase 1/2 (n = 3)

Observational (n = 8)

Prophylaxis (n = 6)

Improper control (n = 7)

Protocol (n = 3)

Duplicate (n = 6)

Full-text article assessed for eligibility (n = 64)

Eligibility

**Study included in qualitative synthesis (n = 6)**

**Study included in quantitative synthesis (n = 6)**

Included

Additional figure 6. Flow chart of study selection of Hidroxychloroquine and Chloroquine in outpatients mild COVID-19

Records identified through databases searching (n = 168)

Medline: n = 102, Embase: n = 18, Clinical Trials: n = 25 and Scholar: n = 23

Manual search (n = 0)

Identification

**Record excluded bases on title and/or abstract** (n = 114)

Screening

Records screened (n = 168)

**Record excluded (n = 51)**

Inpatient (n = 10)

Severe COVID-19 (n = 5)

Prophylaxis (n = 2)

Asymptomatic (n = 2)

Associations (n = 4)

Another outcome (n = 6)

Comment (n = 1)

Meta-analysis (n = 1)

Protocol (n = 2)

RCT phase 2 (n = 10)

Non-randomized (n = 1)

Observational (n = 1)

Improper control (n = 1)

Duplicate (n = 5)

Full-text article assessed for eligibility (n = 54)

Eligibility

**Study included in qualitative synthesis (n = 3)**

**Study included in quantitative synthesis (n = 3)**

Included

Additional figure 7. Flow chart of study selection of Ivermectin in outpatients mild COVID-19

Records identified through databases searching (n = 430)

Medline: n = 329, Embase: n = 51, Clinical Trials: n = 12 and Scholar: n = 38

Manual search (n = 0)

Identification

**Record excluded bases on title and/or abstract** (n = 378)

Screening

Records screened (n = 430)

**Record excluded (n = 44)**

Outpatient (n = 1)

Associations (n = 6)

Another drug (n = 2)

Comment (n = 4)

Interim analysis (n = 1)

Post-hoc (n = 2)

Model (n =2)

RCT phase 2 (n = 1)

Non-randomized (n = 1)

Cohort (n = 8)

Abstract (n = 9)

Review (n = 1)

Duplicate (n = 4)

No results (n = 2)

Full-text article assessed for eligibility (n = 52)

Eligibility

**Study included in qualitative synthesis (n = 8)**

**Study included in quantitative synthesis (n = 8)**

Included

Additional figure 8. Flow chart of study selection of Rendesivir in hospitalized patients with severe COVID-19

Records identified through databases searching (n = 74)

Medline: n = 55, Embase: n = 15, Clinical Trials: n = 2 and Scholar: n = 2

Manual search (n = 1)

Identification

Screening

**Record excluded bases on title and/or abstract** (n = 65)

Records screened (n = 75)

**Record excluded (n = 8)**

Associations (n = 3)

Exploratory clinical trial (n = 2)

Cohort (n = 1)

Abstract (n = 1)

Duplicate (n = 1)

Full-text article assessed for eligibility (n = 10)

Eligibility

**Study included in qualitative synthesis (n = 2)**

**Study included in quantitative synthesis (n = 2)**

Included

Additional figure 9. Flow chart of study selection of Baracitinib in hospitalized patients with severe COVID-19

Records identified through databases searching (n = 430)

Medline: n = 329, Embase: n = 51, Clinical Trials: n = 12 and Scholar: n = 38

Manual search (n = 0)

Identification

**Record excluded bases on title and/or abstract** (n = 378)

Screening

Records screened (n = 430)

**Record excluded (n = 37)**

Associations (n = 4)

Protocol (n = 3)

Interim analysis (n = 1)

Post-hoc (n = 3)

RCT phase 2 (n = 3)

Short follow-up (n = 1)

No control or improper (n = 4)

Cohort (n = 2)

Abstract (n = 6)

Review (n = 1)

Duplicate (n = 10)

Full-text article assessed for eligibility (n = 51)

Eligibility

**Study included in qualitative synthesis (n = 14)**

**Study included in quantitative synthesis (n = 14)**

Included

Additional figure 10. Flow chart of study selection of Tocilizumab in hospitalized patients with severe COVID-19

**
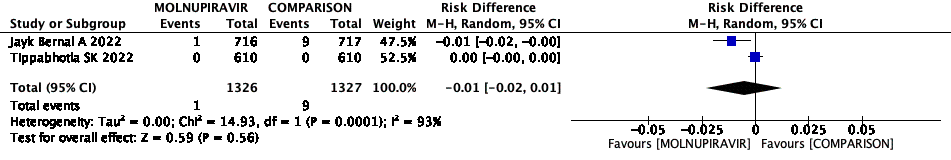
**

Additional figure 11. Effect of Molnupiravir compared to control on mortality of outpatients with mild COVID-19

**
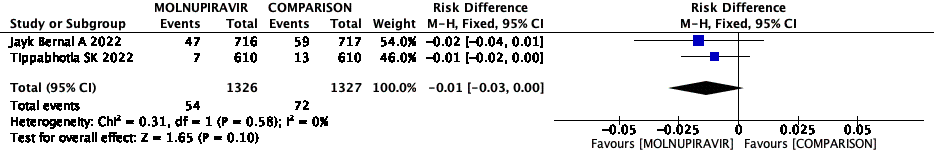
**

Additional figure 12. Effect of Molnupiravir compared to control on hospitalization of outpatients with mild COVID-19

**
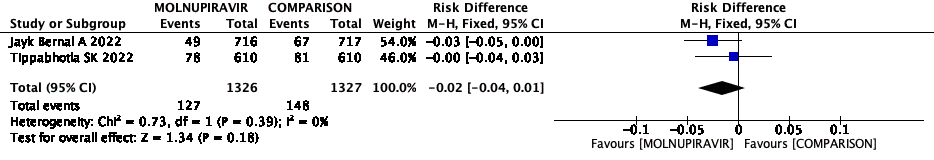
**

Additional figure 13. Effect of Molnupiravir compared to control on serious adverse events in outpatients with mild COVID-19

**
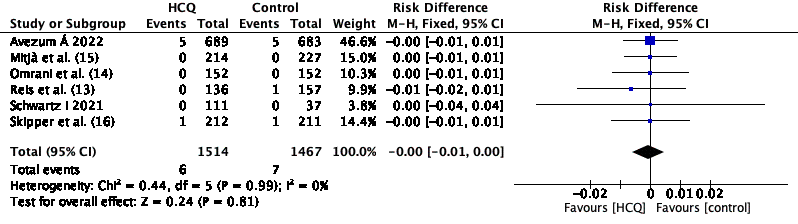
**

Additional figure 14. Effect of Hidroxychloroquine and Chloroquine compared to control on mortality of outpatients with mild COVID-19

**
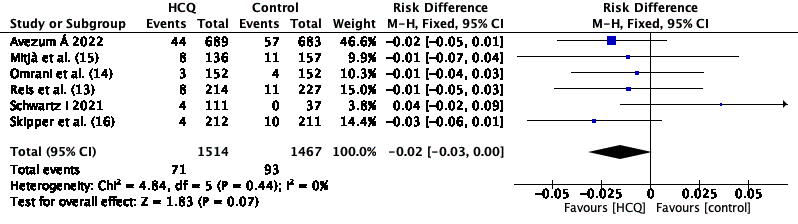
**

Additional figure 15. Effect of Hidroxychloroquine and Chloroquine compared to control on hospitalization of outpatients with mild COVID-19

**
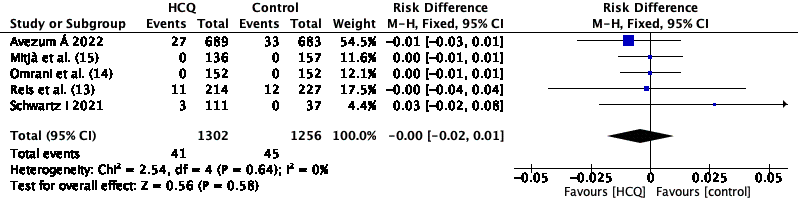
**

Additional figure 16. Effect of Hidroxychloroquine and Chloroquine compared to control on serious adverse events in outpatients with mild COVID-19

**
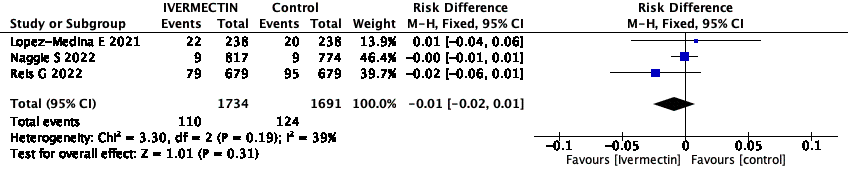
**

Additional figure 17. Effect of Ivermectin compared to control on hospitalization of outpatients with mild COVID-19

**
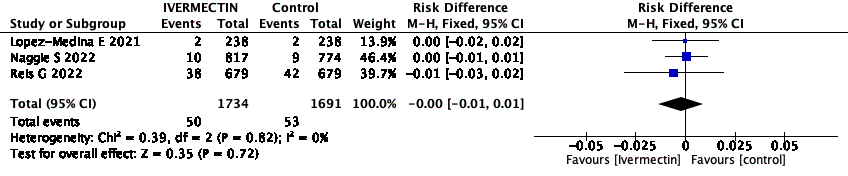
**

Additional figure 18. Effect of Ivermectin compared to control on serious adverse events in outpatients with mild COVID-19

**
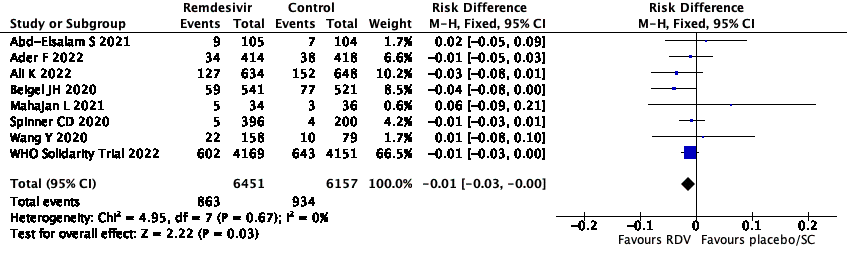
**

Additional figure 19. Effect of Remdesivir compared to control on mortality of hospitalized patients with severe COVID-19

**
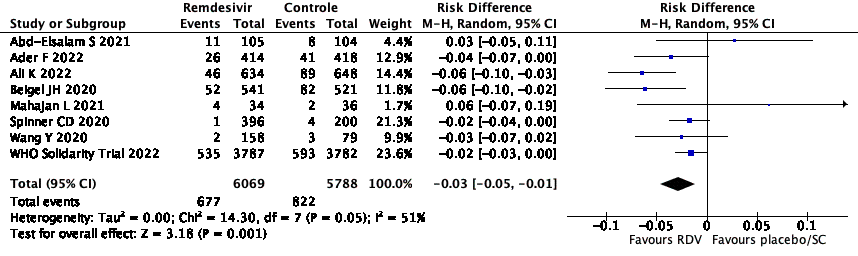
**

Additional figure 20. Effect of Remdesivir compared to control on mechanical ventilation of hospitalized patients with severe COVID-19

**
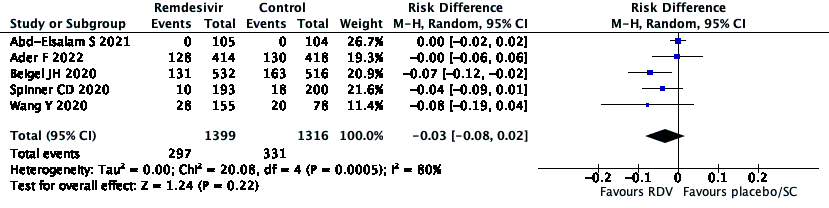
**

Additional figure 21. Effect of Remdesivir compared to control on serious adverse events in hospitalized patients with severe COVID-19

**
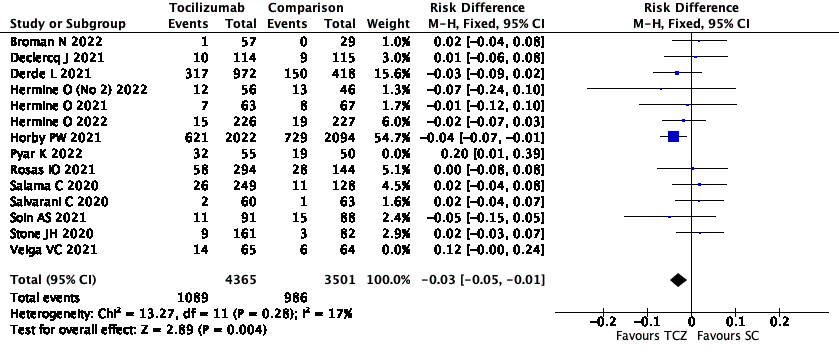
**

Additional figure 22. Effect of Tocilizumab compared to control on mortality in hospitalized patients with severe COVID-19

**
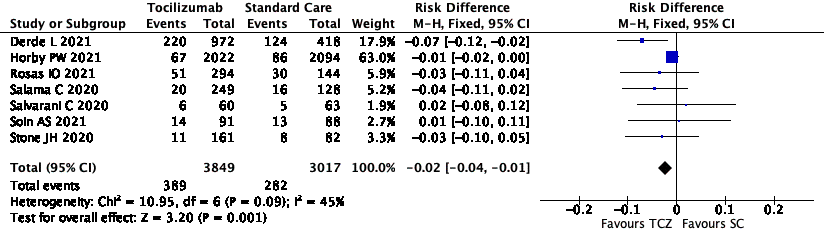
**

Additional figure 23. Effect of Tocilizumab compared to control on mechanical ventilation in hospitalized patients with severe COVID-19

**
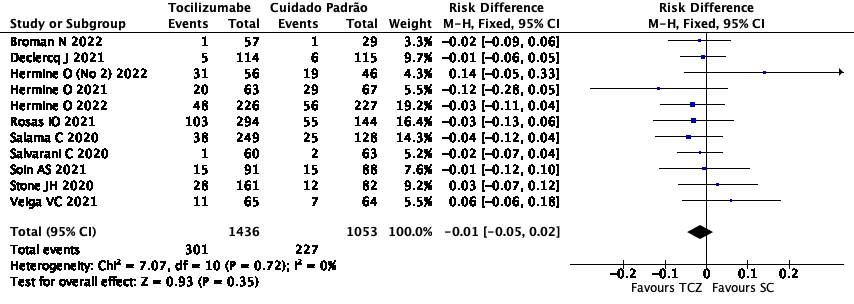
**

Additional figure 24. Effect of Tocilizumab compared to control on serious adverse events in hospitalized patients with severe COVID-19


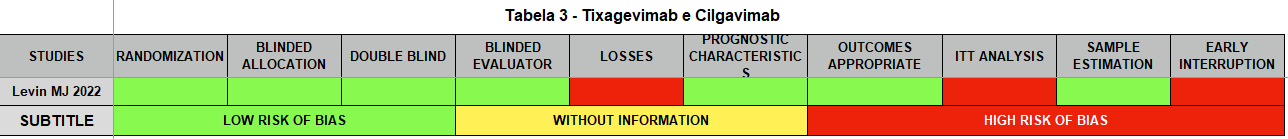


Additional figure 25. Risk of bias assessment for the study of Tixagevimab + Cilgavimab in COVID-19 pre-exposure prophylaxis


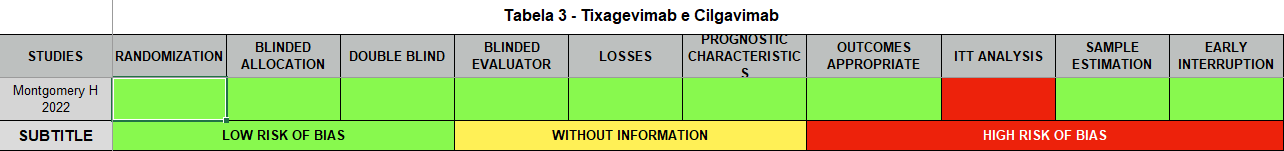


Additional figure 26. Risk of bias assessment for the study of Tixagevimab + Cilgavimab in outpatients with mild COVID-19


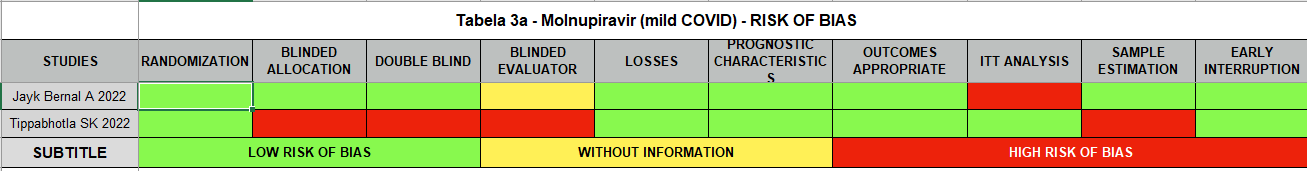


Additional figure 27. Risk of bias assessment for the studies of Molnupiravir in outpatients with mild COVID-19


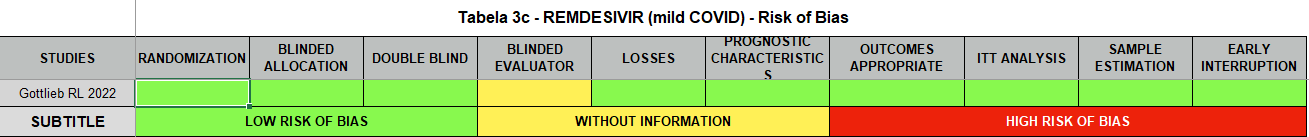


Additional figure 28. Risk of bias assessment for the study of Remdesivir in outpatients with mild COVID-19


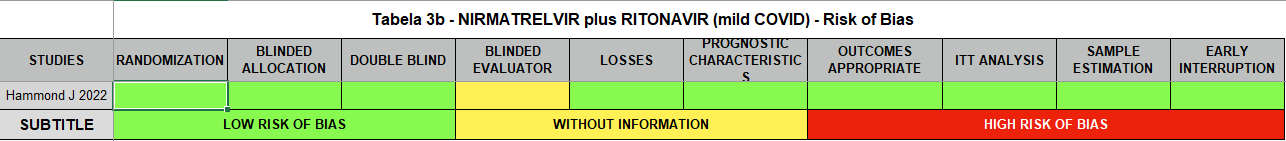


Additional figure 29. Risk of bias assessment for the study of Nirmatrelvir plus Ritonavir in outpatients with mild COVID-19


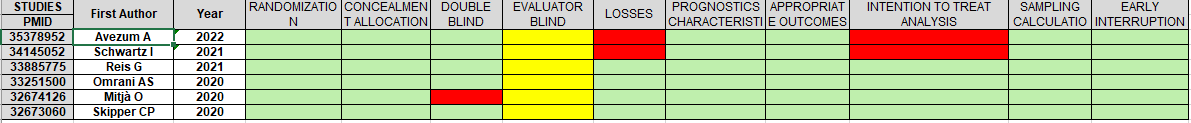


Additional figure 30. Risk of bias assessment for the studies of Hidroxychloroquine and Chloroquine in outpatients with mild COVID-19


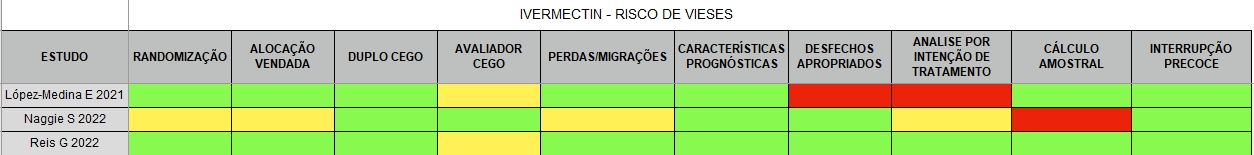


Additional figure 31. Risk of bias assessment for the studies of Ivermectin in outpatients with mild COVID-19


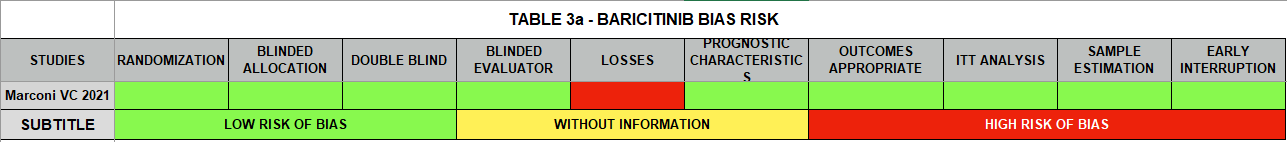


Additional figure 32. Risk of bias assessment for the study of Baricitinib in hospitalized patients with severe COVID-19


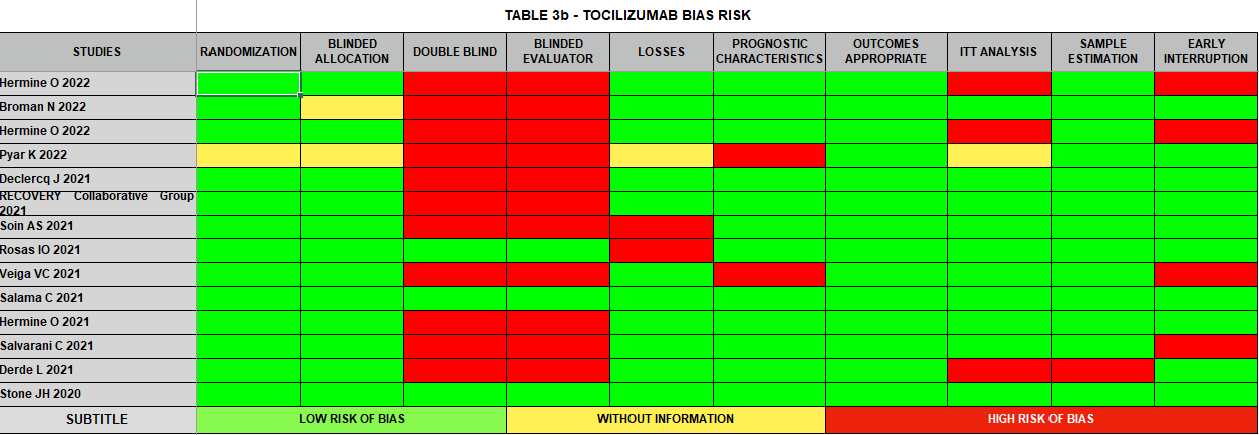


Additional figure 33. Risk of bias assessment for the studies of Tocilizumab in hospitalized patients with severe COVID-19
